# Cell – extracellular matrix interaction in glioma growth. *I* *n silico* model

**DOI:** 10.1515/jib-2020-0027

**Published:** 2020-12-14

**Authors:** Vladimir Kalinin

**Affiliations:** R&D Sector, Techno-Modeling Arts Ireland, Unit 8, Cul na Raithe, A91K8KR, Louth, Ireland

**Keywords:** cell adhesion, cell–ECM stress, extracellular matrix, glioma, *in silico* model, multicellular tumor spheroid, subtumours

## Abstract

The study aims to investigate the role of viscoelastic interactions between cells and extracellular matrix (ECM) in avascular tumor growth. Computer simulations of glioma multicellular tumor spheroid (MTS) growth are being carried out for various conditions. The calculations are based on a continuous model, which simulates oxygen transport into MTS; transitions between three cell phenotypes, cell transport, conditioned by hydrostatic forces in cell–ECM composite system, cell motility and cell adhesion. Visco-elastic cell aggregation and elastic ECM scaffold represent two compressible constituents of the composite. Cell–ECM interactions form a Transition Layer on the spheroid surface, where mechanical characteristics of tumor undergo rapid transition. This layer facilitates tumor progression to a great extent. The study demonstrates strong effects of ECM stiffness, mechanical deformations of the matrix and cell–cell adhesion on tumor progression. The simulations show in particular that at certain, rather high degrees of matrix stiffness a formation of distant multicellular clusters takes place, while at further increase of ECM stiffness subtumors do not form. The model also illustrates to what extent mere mechanical properties of cell–ECM system may contribute into variations of glioma invasion scenarios.

## 1 Introduction

Glioblastoma multiform (GBM) is one of the most lethal types of human cancers with a median survival time slightly above a year [1]. Rapid proliferation and aggressive invasion into surrounding normal tissue is a hallmark of GBM. This ability of long range infiltration of the primary tumor cells through brain tissue even in the absence of distant metastases, makes GBM a primary target of many research projects including *in vitro* studies and computer models [1], [2], [3].

It has been found [4], [5] that purely mechanical characteristics of glioma microenvironment may play pivotal role in the invasion process. The importance of cell–extracellular matrix (ECM) interaction and the role of matrix density, elasticity and porosity have emerged as a particularly strong regulators of migration and proliferation rate of malignant brain tumor cells. There are number of observable trends reported by different authors [3], [7], [8], [9].

The invasive distance was found to be typically biphasic function of ECM pore size increasing in the range of small pore sizes and decreasing at wider pores [7], [10]. The maximum invasiveness is shifting towards wider pore size with increase of ECM stiffness [7]. Glioma cells exhibit increase of cell motility in narrow channels and may demonstrate ability of amoeboid squeezing through narrow pores with sizes below their nuclear diameter [11], [12]. There are certain difficulties in generalization of those observations in order to reduce them to simple dependences on ECM porosity or density. Multiple attributes of the matrix microstructure, such as channel configuration (width/length ratio), fiber thickness, fiber alignment and crosslink density may significantly affect cell invasiveness and should be taken into account in data analysis [5], [7], [13], [14].

An increase of matrix density, which affects its stiffness as monotonic function, leads typically to formation of denser spheroids with increased invasiveness [15], [16], [17]. ECM stiffness has been found to influence strongly glioma invasiveness [4], [5], [6], [7], [18], [19], [20]. On the other hand, highly elastic glioma cell lines demonstrate more aggressive behavior when compared with stiffer cells cultured in similar substrate [21]. Based on the reported results, one can conclude that the ratio of ECM- to -cells stiffness has a strong modulatory effect on glioma cell invasion and migration characteristics. Typically, a system of ‘soft cells in stiff matrix’ demonstrates high invasive potential.

Nevertheless, about one quarter of the studied cell lines exhibit mechanosensitivity to ECM stiffness different from that typically observed, demonstrating biphasic function of rigidity-dependence [19] or rigidity-insensitive behavior [5]. Interestingly, these cells insensitive to the matrix stiffness exhibited the highest invasive capacity even in soft matrigel 3D cultures [5]. Therefore, in spite of the general trends every case is unique regarding the behavior of each specific glioma cell line. It was also shown in [4], [19] that glioma cell behavior, being migratory dependent on purely mechanical effects of stresses and ECM structure, may at the same time be mediated though more complicated pathways involved in glioma invasion process.

The role of ECM stiffness in glioma growth can be partially explained, as it conditions mechanical stresses in tumor microstructure, which has dramatic influence on the growth dynamics. The mutual influence of tumor cells and the microenvironment through the mechanical stresses can modulate migration and therefore invasiveness of tumor [22], [23], [24] and also regulate proliferation and apoptosis of tumor cells [25], [26]. Despite the observed correlations of tumor growth with the stresses and cell–ECM rheology, the precise role of the microenvironment in this process is still not well studied [3], [4], [9], [27].

Not only values, but a special form of the local stresses distribution across tumor border provides its progression. That particular distribution has been observed in glioma MTS growth where pushing and traction forces have been measured along MTS radius across its border [28]. The authors suggested that the traction forces may cause direct migration through tracks formed by ECM alignment that further promotes elongation of tumor cells along the traction line. In so doing, the growing MTS exerts an outward ECM pressure at the distances greater than its own size. Similar “pushing-and-pulling” scenario across the edge of MTS core has been demonstrated on murine colon carcinoma cell line by Kopanska and colleagues [29]. The authors showed ECM radial contraction towards MTS surface and tangential alignment of the gel fibers close to the surface. Generally, the presence of traction forces exerted by cells in a 3D matrix was observed in number of works e.g. [15], [29], [30] and measured in the range of 100 and 250 pN.

One of the key questions associated with these observations would be to what extent the observed scenarios are specific to particular cancer cells or particular type of ECM structure? Regarding glioma cells, it was shown that different cell lines may form substantially different invasive patterns [6], [31], [32], resulting from different invasive strategies. Therefore, generalization of the results obtained for a certain cell line to all glioma cells may be seriously restricted by significant variation of mechanobiological properties between different cell lines. One such difference is elasticity of different glioma cell lines, as shown by Louca and colleagues [21].

The relative contributions of stress or morphology related attributes into tumor progression are difficult to rectify in experiments, as they are often interconnected, do not allow independent variation and may affect other physical characteristics of cell–ECM system, such as focal adhesion and matrix stiffness [6], [19]. *In silico* models, being free from these limitations may seriously assist in further analysis of glioma invasiveness. Computer simulation can be particularly efficient in study of purely mechanical properties of tumor, based on clear and well known principles.

Rheological tumor properties have been modeled initially as a single homogeneous continuum [33] and later within multi-phase approach [34], [35], [36], [37]. A general separation of the tension zone in the center of a tumor spheroid from the zone of compression in the spheroid periphery has been shown within the approach of elastic incompressible continuum [33]. The only source of stress in the model was cell proliferation resulting in tumor expansion. The multi-phase models are generally focused on interaction between the phases. Cells inevitably represent one of the phases, while ECM most of the time represents another one.

Two elastic incompressible phases interact in the model of Ngwa and Agyingi [35]. Spherically symmetric cell agglomeration – phase I is expanding and exerting pressure on an external medium – phase II. In return the phase II offers certain resistance to the growing tumor, which is relatively small comparing with the stress effect related to its growth. Nevertheless the resistance of the surrounding medium clearly influenced the saturation size of the tumor. It was also concluded that the conditions of nutrient diffusion may impose significant limitations to the nutrient supply and therefore influence the tumor saturation size.

Further to demonstration of experimental evidence of cell compressibility [38], [39] this property has been included into *in silico* models. One such approach was used in Ref. [36], where cells modeled as elastic and compressible aggregation with Poisson factor *ν* = 0.2. ECM was modeled in the work as a rigid structure and this model was intended to study the growth of encapsulated MTS under external stress. The calculated radius of tumor growing in confined environment as function of time was in good correlations with experimental results of Ref. [38], as well as with the outcome of their own *in silico* model based on assumption of elastic extracellular scaffold. Therefore, the assumptions of rigid [36] and elastic [38] ECM did not show significant difference in simulated results. However, another numerical study [37] demonstrated actual difference in tumor development for these two alternatives. The cells have been modeled as uncompressible fluid, while ECM considered as a porous elasto-visco-plastic or rigid solid material [37]. The simulations based on these alternatives showed faster growth of tumor in elastic matrix in comparison with the case of rigid ECM, as the deformable ECM changes its porosity under the pressure exerted by growing tumor. The exercise demonstrated the importance of ECM deformability as a critical factor of tumor growth. The simulation results are in line with *in vitro* study of human colon, murine mammary cancer and rhabdomyosarcoma cells [39], where the MTS growth was insensitive to ECM stiffness up to a certain threshold when exhibited a sharp step down in the final spheroid sizes. Regarding glioma growth, however laboratory studies generally show an inverse system of ‘soft cells in rigid matrix’ as a condition for most aggressive tumor growth [4], [5], [6], [7], [17], [18], [19], [20], [21]. This comparison stresses the point that mechanobiological theory of tumor progression is still in its infancy and cell–ECM interaction scenarios need further study.

It was found that glioma cells follow “Go or Grow” (GoG) principle as common rule for growing tumor. The GoG concept proposes that cells are unlikely exhibiting both proliferation and migration concurrently and is based on observations of astrocytoma cells rarely dividing while moving [40]. While it was clearly demonstrated in further observations [41] that proliferation and migration of glioma cells are inversely correlated processes, the factors triggering switch of cell phenotype are still not quite clear. A shortage of nutrients is generally considered as factor of GoG switch, causing metabolic stress in glioma cells [42]. In particular, there are many results showing oxygen concentration as critical parameter for the transition of cell phenotype. The “hypoxic” concept of switching is widely exploited in models and analyses of glioma and general tumor growth [43], [44], [45], [46], [47]. Another nutrient often considered as critical for GoG switch is glucose [48]. Various microenvironmental conditions or a threshold of high cell density were also assumed to trigger the change of cell phenotype [40], [49], [50], [51]. And finally some factors particularly specific to glioma, such as decrease of astrocytes density on tumor periphery below certain level [52] may trigger the transition.

The nutrient based transition criteria are mutually connected, as the nutrient profiles exhibit similar shapes. The profiles are generally determined by strong concentration gradients along the MTS radius resulting from diffusion into the area of high cell density and therefore increasing nutrient consumption [53]. The environmental transition triggers do not have obvious connection to nutrient based thresholds and therefore represent different model of tumor growth. Completely apart from the considered above switch conditions is assumption that the phenotype transition is purely stochastic event [54]. This approach is justified to certain extent by poor current knowledge of the actual conditions leading to cell phenotype transition and the absence of a general concept for phenotype triggering criteria. The switch criteria of different nature may generate substantially different invasive scenarios [44], [53].

Another important factor of GoG dichotomy is probably the overlapping of phenotype characteristics. The main question here is whether migration and proliferation states exclude each other completely, strictly “Go-or-Grow”, as considered in Refs. [45], [54] or, if not then to what extent these inversely correlated features can overlap to be exhibited at the same time by the same cell, as in Ref. [46]. Two polar cases have been modeled in Ref. [44] where the case of “Go-and-Grow” features overlapped completely was compared with pure “Go-or-Grow” case. The results show “Go-or-Grow” strategy as more efficient in terms of glioma progression. However, a comparative analysis of the simple polar scenarios and the cases of partial ‘Go and Grow’ overlapping is still not carried out.

Generally in glioma growth the GoG dichotomy has significant influence on width of the invasive zone and tumor expansion dynamics, which is expected to be sensitive to transition characteristics including transition times and thresholds [44], [50], [53], [54]. The research results demonstrate how important role cell phenotype switch plays in tumor progression and how little at the same time we know about the criteria and characteristics of this process.

Another important factor influencing the growth of glioma is cell adhesion–repulsion mechanism [34], [50], [55], [56], [57], [58]. The interplay between cell phenotype transition dynamics and cell–cell interaction determines to great extent the shape and speed of invasive glioma front and its growth characteristics [44], [51], [55].

Pham and colleagues [51] considered cell–cell interactions by introducing density-dependent regulation of migration/proliferation dichotomy in a reaction–diffusion model and found significant influence of this interaction on the dynamics of tumor front. Number of simulations based on continuous and discrete models showed that glioma invasiveness is generally reduced when cell–cell attractive interactions grow up [58], [59], [60], [61]. These results were consistent with experimental data.

Cell–cell adhesion–repulsion mechanism also plays important role in formation of subtumors [55], [56], [57]. Cell–cell adhesion is responsible for formation of multicellular groups, strands and clusters forming at the tumor interface and growing away from the main tumor mass. It was shown *in silico* by Frieboes et al. [55] that subtumors are being formed by all cells of the tumor surface and not as a result of hyper-proliferation of a distant single cell [55]. This result demonstrates that cell motility is probably not the key factor in the formation of subtumors, when a single highly motile cell could invade far into nutrient-rich area and hyper-proliferate there. The actually observed scenario is that specific stress distribution forms hydrostatic pushing forces on the front providing all cells drift in a bias towards the invasion front regardless of their phenotype. This shows again that the distribution of stresses and therefore viscoelastic properties of tumor components including cell aggregation and ECM are strong modulators of glioma progression.

Summarizing the major outcome of the previous works one can conclude that variation of purely mechanical properties of ECM, such as its microstructure, deformability and compressibility, may significantly affect tumor growth. The experimental observations, briefly presented here and mostly dedicated to glioma, show dramatic influence of stress factor on its growth. The mechanism of this influence is presumably that stress can potentially modify the structure and density distribution of the major tumor components – ECM and cell aggregation, which in turn modulates tumor growth characteristics.

Overall, rheological properties of compressible tumor components, cell adhesion and cell phenotype transition are probably the key combination of mechanobiological attributes determining the regime and characteristics of glioma progression. All these attributes are included into the *in silico* model developed in the current work.

One of the objectives was to build a model demonstrating scenarios of cell–ECM interaction driven by mere mechanical principles of continuum that can be used as a basis in analyses of tumor growth processes observed in laboratory tests or a clear first approximation for further simulations of glioma variability, involving signaling pathways.

Another objective of the work was clarification of basic invasive scenarios in the frame of the model and their sensitivity primarily to matrix rheology and oxygen supply.

## 2 *In silico* model

We consider growing MTS as a composite system comprising two basic components: tumor сells and ECM. The interstitial fluids (IFs) are also part of this composite system. As first approximation, the interstitial fluids are assumed to represent an incompressible continuum, traveling through the matrix and cellular content without any resistance being always part of these two basic components.

Cell aggregation, as well as ECM, normally exhibits multiple voids [62]. Therefore, the “dry content” of the basic fractions is porous and compressible. IFs fill the voids of the cell aggregation and porous ECM scaffold, but theoretically can be squeezed out of these system components under external pressure, as they both are fully transparent for IFs. The details of IFs dynamics are not considered in the model.

In mathematical approximations, we formally consider only two system components: fluidized cell aggregation and fluidized ECM, assuming the sum of their volume fractions equal to 1.

ECM is considered to be purely elastic compressible scaffold, while the cell aggregation exhibits viscoelastic properties and also effects related to cell–cell adhesion, see the details below.

The model includes three cell phenotypes:

– Proliferative cells that can proliferate and have low or zero motility;
– Hypoxic cells that have low or zero proliferation rate and exhibit high motility;
– Necrotic cells, which are inert-do not exhibit motility and do not proliferate.

The transition between cell phenotypes is determined by two factors: concentration of oxygen as a switch threshold and characteristic transition times. Proliferative- to -hypoxic and inverse transitions are considered in the model along with hypoxic- to -necrotic switch. Removal of necrotic cells is not taken into account as not important process within current simulation times.

The model is based on continuum approximation. Some of the dynamic parameters of the model are being further obtained under the following assumptions:

(a) Cancer cells comprise incompressible viscoelastic spheres. In general case, the cell colony may include multiple voids, having relatively low average cells concentration. A massive cell colony is isotropic visco-elastic compressible agglomeration of spheres.
(b) Extracellular Matrix comprises isotropic, compressible, linear elastic, porous material. Cells proliferation and their motion through this scaffold produce elastic stresses in ECM, which may change the transport conditions for these cells.
(c) Spherically symmetric simulation domain contains two basic components: cells and ECM, and does not present any phenomenologically determined spatial subdivision of its physical content into different parts (e.g. free moving boundaries with preset shedding rates out of them). Only initial conditions determine a clear boundary of the initial tumor spheroid. No further definitions of the border between the core spheroid and ECM are imposed and the distribution of partial densities in the composite system is formed in self-consistent simulation.
(d) The cells exert attractive and repulsive forces on each other depending on intercellular distances. The lower limit of cell concentration in the colony is determined by the critical distance between them. Beyond this threshold cells cannot sense or forcibly affect each other.
(e) The total stress distribution in the composite system follows iso-strain approximation. This intercellular force field together with ECM stress gradients induces cells motion tending to minimize the total potential energy of the system.
(f) Cell motility is modeled through their diffusion. The diffusion rate is specific for each cell phenotype.

### 2.1 Basic processes in details

#### 2.1.1 Attraction and repulsion of cells, cell compressibility

There are rather limited data available in literature on compressibility measurements for cell aggregation, as a separate phase independent of ECM. A combination of modeling and experimental measurement of cellular aspiration in a micropipette gives typical Poisson’s ratios of *ν*_*C*_ = 0.3–0.4 [63] in contrast to typically assumed value of 0.5 [64]. This may result from multiple discontinuities in cell clusters that could provide certain potential for compression. Therefore, apart from single cell compressibility, a cell colony should represent rather compressible aggregation. This presumption is confirmed by *in vitro* studies of MTS growth [40], which showed that general compressive stress may lead to an increase of cellular packing density. However, even a study of single cell size variations under pressure showed substantial compressibility. Melanoma cells for instance exhibit *K*_*C*_ ≈ 2.5 kPa [38]. The authors demonstrated variation of the available data for bulk moduli of different cancer cells in the range 2.5–30 kPa. At the same time, the other sources show extremely low general cell compressibility *K*_*C*_ = 2.5 × 10^9^ Pa [65]. Having no specific data on a single glioma cell compressibility we are not actually bound to this particular characteristic. The current model does not consider dense cell aggregations behaving as a homogeneous substance with bulk modulus close to same of a single cell. The porosity of glioma cell aggregation always contributes into its compressibility. The correspondent rheological parameters are being used in the model, see section 2.3 “Choice of Parameters”. This compressible cell colony can be described in first approximation as accumulation of visco-elastic incompressible spheres. Each sphere consists of homogeneous elastic medium with a smooth boundary (similar to that assumed in Hertz or Sneddon models) covered with a layer of membrane protrusions providing cell–cell adhesion similar to “brush” models [66].

**Table 1:** Mobility and random motility of Glioma Cells.

Characteristic	Value	Source
Collagen permeability, ***k***	10^−12^ m^2^	[85]
Dynamic viscosity of cancer cells, *μ*	300 Pa s	[83]
Mobility of cells in collagen, ***M***	2.9 × 10^−4^ mm^2^/(Pa d)	Current. Calc.
Hypoxic cells, *D*_*m*_	2.0 × 10^−2^ mm^2^/day	[47]
Proliferative cells, *D*_*p*_	*D*_*p*_ = *D*_*h*_/10	

**Table 2:** Rheological parameters.

Parameter	Value	Source
Poisson ratio for collagen, *ν*_ECM_	0.13	[89]
Young’s modulus for collagen, *E*_ECM_	0.5–2.5 kPa	[89]
Bulk modulus for collagen, *K*_ECM_	0.3–1.13 kPa	Current. Calc.^a^
Poisson ratio for cells, *ν*_C_	0.4	[63]
Young’s modulus for cells, *E*_C_	300 Pa	[91]
Bulk modulus for cells, *K*_C0_	500 Pa	Current. Calc.^a^

^a^Assessment is based on the values presented in the table.

**Table 3:** Oxygen transport and consumption.

Characteristic	Value	Source
Oxygen diffusion coefficient *D*_O2_	86.4 mm^2^/day	[94]
Oxygen diffusion coefficient *D*_O2_^a^	40.0 mm^2^/day	Used for comparison purposes
Oxygen uptake **for proliferative cells *C***_**p**_		
**α**_**p**_ [(mm^3^/Cell)mg/(L*Day)]	**α**_**p**_ = 1.38 × 10^−2^	[47]
Oxygen uptake **for hypoxic cells C**_**m**_ **: α**_**h**_	**α**_**h**_ ** = α**_**p**_ **/5**	[47]
**Critical oxygen concentrations**		
Threshold to hypoxia	*ξ*_*h*_ = 1.0 mg/L	[99]
Threshold to necrosis	*ξ*_*n*_ = 0.8 mg/L	[99]

**Table 4:** Proliferation and phenotype transition times.

Characteristic times	Value	Source
Proliferative to hypoxic phenotype	*τ*_*ph*_ * = *1 h	[47]
Hypoxic to proliferative phenotype	*τ*_*hp*_ * = *96 h	[47]
Hypoxic to necrotic phenotype	*τ*_*hn*_ * = *32 h	[52]
Proliferative cells doubling time	*τ*_*p*_ * = *24 h	[47]
Hypoxic cells doubling time	*τ*_*m*_ * = *48 h	[47]
Hypoxic cells death time	*τ*_*n*_ * = *48 h	[47]

A theoretical maximum *C*_max_ of cell density is fixed and determined by the volume of a single cell. Taking the figure *V*_Cell_ = 1200 μm^3^ of the EMT6/Ro rat brain tumor cell volume [67] as typical, we can assess *C*_max_ as 8.3 × 10^5^ mm^−3^. This dense cell packing does not include any fluids or ECM. The volume fraction of cells in MTS can be defined as Φ_*C*_ *= C*_tot_/*C*_max_, where the concentration C_tot_ includes all types of cells.

Separation distances between sphere centers determine the local cell density. An important characteristic of that cellular system is intercellular equilibrium distance *r*_eq_. In the absence of any other attractant but the cells, they should move in a way that minimizes the potential energy between them. Cells may move towards one another at *r* > *r*_eq_ due to adhesion. In case of compression *r < r*_eq_, the cells should move due to increasing repulsive forces towards the area offering more free space. Another important parameter for the system of interacting spheres is the cut off distance *r*_∞_, which is the minimum separation distance between sphere centers when cells lose completely their mutual connection. In other words, at *r > r*_∞_ any two cells in the model do not affect each other. At uniform cell distribution this situation corresponds to cell density *C*_cell_ *< C*_∞_.

#### 2.1.2 Parameters of cell–cell interaction

As long as the current numerical model operates with cellular continuum all critical parameters of the discrete cell agglomeration can be interpreted in the form of cell concentrations. An equilibrium cell concentration *C*_eq_ corresponds to equilibrium separation distances between sphere centers *r = r*_eq_, so that at cell densities *C*_cell_ *> C*_eq,_ the cell agglomeration undergoes expansion and otherwise at *C*_cell_ *< C*_eq_ – contraction.

Experimental results for glioma MTS [70] show the inner quasi-stationary cell density in dynamically growing spheroid about *C*_eq_ = 4 × 10^5^ cells/mm^3^, which agrees well with their assessment of *C*_eq_ based on the volume *V*_*C*_ = 1200 μm^3^ of EMT6/Ro tumor cells. It was assumed that cell spheres in the colony at their equilibrium density are in direct contact with maximum number of neighbors, but still not stressed by these neighbors. Slightly lower equilibrium volume fraction of 0.39 for tumor cells was taken in Ref. [69] for a model of GBM growth, which corresponds to *C*_eq_ = 3.2 × 10^5^ cells/mm^3^. We used value *C*_eq_ = 3.5 × 10^5^ cells/mm^3^ in the current work. The Force–Distance (*F*–*D*) curves for human glioma cells show maximum attraction force at ∼3 μm separation [70]. This force exhibits ten-fold decrease at separation distances about 20 μm and at 50 μm the cells are regarded as detached [70]. Assuming 20 μm separation between cell surfaces is a technical cut off, one can obtain the cut off distance of *r*_∞_ **≈** 33 μm between the centers of spherical cells once *V*_Cell_ = 1200 μm^3^. Then the average cut off concentration of spherical cells can be assessed as *C*_∞_ *= *2.8 × 10^4^ cells/mm^3^, while maximum attraction force should correspond to *r*_att_ ≈ 16.2 μm and *C*_att_ ≈ 2.35 × 10^5^ cells/mm^3^.

The model of spheres is used here only to assess rheological parameters of a cell colony. For the purpose of numerical simulation, the cell colony is described as visco-elastic continuum with density distribution *C*(*r*) and ability to build inner positive pressure, as well as inner contractile forces. Having determined the key parameters *C*_∞_, *C*_eq_ and *C*_max_, we can further consider cell colony as visco-elastic continuum with bulk modulus *K*_*C*_ defined as:

$$K_{C}\left(C_{\text{tot}},\;C_{\text{eq}}\right)=C_{\text{tot}}\frac{d\sigma_{C}}{dC_{\text{tot}}}$$

where *C*_tot_ is total cell concentration including all cell phenotypes.

This further gives linear approximation for intercellular stress *σ*_*C*_

(1) $$\sigma_{C}=K_{C0}\left(\frac{C_{\text{tot}}}{C_{\text{eq}}}-1\right)\text{,}$$

which is feasible within limited vicinity of equilibrium value *C*_eq_, when *C*_tot_ *<*< *C*_max_. Here, the constant *K*_*C*0_ is the bulk modulus at equilibrium cell concentration *C*_tot_ *= C*_eq_. Obviously, this linear approach is not valid at high local raise of cell concentrations *C*_tot_ *∼ C*_max_. It is reasonably assumed that tumor expansion process has quasi-static character, when *C*_tot_ does not grow too high above local equilibrium density determined by cell interactions along with the stress of extracellular matrix. This assumption has been justified by further MTS growth calculations. The other works e.g. [68], [69] also show smooth gradients of cellular density in GBM tumor, which typically correspond to quasi-static growth character and smooth time isolines.

In the case of volume expansion *C*_tot_ ≤* C*_eq_ the expression (1) yields growing negative stress. This corresponds to an increase of intercellular distances at decreasing cell concentration.

As determined above, maximum attraction force *f*_max_ between the two cells is achieved at their homogeneous concentration *C*_att_ ≈ 2.35 × 10^5^ cells/mm ^3^. And *F*–*D* curve for human glioma cells shows *f*_max_ ∼ 430 pN at this maximum [70]. Further, assuming the number of neighboring cells roughly equal to 12, as kissing number for close-packing of equal spheres [71] and the surface area of a single sphere *S*_cell_ ≈ 550 μm^2^ for *V*_Cell_ = 1200 μm^3^, we can assess the maximum contractile stress as max *σ*_att_ ∼ 10 Pa. To satisfy contractile force characteristics at low cell densities another term *γ* should be introduced in (1) for *C*_tot_ ≤* C*_eq_, so that

(1a) $$\sigma_{C}=\gamma K_{C0}\left(\frac{C_{\text{tot}}}{C_{\text{eq}}}-1\right)\text{,}$$

where

$$\gamma =\left\{ \begin{array}{ll} 1 & C_{\text{tot}}\geq C_{\text{eq}}\\ \frac{27}{4}\frac{\sigma_{\text{att}}}{K_{C0}}\frac{C_{\text{eq}}}{{\left(C_{\text{eq}}-C_{\infty }\right)}^{3}}{\left(C_{\text{tot}}-C_{\infty }\right)}^{2} & C_{\text{eq}}>C_{\text{tot}}\geq C_{\infty }\\ 0 & C_{\text{tot}}<C_{\infty } \end{array}\right.$$

The approximation of contractile stresses at *C*_tot_ <* C*_eq_ is based on *F*–*D* curve for glioma cells and does not depend on bulk modulus *K*_*0*_ for the cell aggregation. It satisfies characteristic parameters and general behavior of the curve.

#### 2.1.3 Invasive cells motility

The motility of invasive cells is described as diffusion in the model and follows GoG concept. Proliferative cells can transition into hypoxic cells within characteristic time *τ*_pm_ if the local concentration of oxygen drops below hypoxic level. In turn, hypoxic cells can transition to necrotic cells within characteristic time *τ*_mn_ since the local oxygen concentration decreases further below necrotic barrier. At the same time, hypoxic cells can transition back to proliferative phenotype once the local oxygen concentration exceeds the transition barrier. A two switch approximations have been modeled in the present work.

*Stiff switch approach* strictly follows GoG rule, where all hypoxic cells exhibit invasive migration and are assumed non-proliferative, while all proliferative cells are non-invasive.

*Overlapping switch* allows certain motility of proliferative cells and attributes some proliferation to hypoxic cells as well. It was further shown in simulations that the “*Overlapping switch*” provides better agreement with experimental results. The switch parameters used for the calculations are presented in “*Choice of Parameters*” section.

The third phenotype-necrotic cells are totally inert. Total cell density is a sum of the three partial cell concentrations:

$$C_{\text{tot}}=C_{p}+C_{m}+C_{n}$$

where

*C*_*p*_ – concentration of proliferating cells;

*C*_*m*_ – concentration of hypoxic cells;

*C*_*n*_ – concentration of necrotic cells

#### 2.1.4 Stresses in cells–ECM composite

MTS growth builds up stress gradients in the cells–ECM composite. This process conditions a self-consistent dynamics in the cell aggregation that represents motion of individual cells, as well as ECM displacements in the way that minimizes these stress gradients.

According to (1a), the local stress *σ*_*c*_ in the cell colony can be negative due to cell–cell adhesion, which may cause local contraction of the cell aggregation. Contrariwise, a local increase of total cell concentration above *C*_eq_ produces an increase of the local stress followed by expansion of the cell colony. ECM displacements make their own contribution into the distribution of total stress in the system *σ*_tot_ through building up stresses in ECM *σ*_ECM_. Cell invasion into ECM, as well as general growth of cell colony due to their proliferation is taken into account as a volumetric force that produces displacements of ECM, see equation (5).

The hydrostatically conditioned cell motion is described in the model as a drift with velocities *v*_*D*_ proportional to the total stress gradient (2), which is typical for continuum models of avascular tumor [72], [73].

(2) $$v_{D}=-M\frac{\partial \sigma_{\text{tot}}}{\partial r}$$

*σ*_tot_ – total stress in the system. *M* is cell mobility that reflects to certain extent the effects of cell adhesion. As long as cell affinity to each other and to the matrix contributes to their viscosity one should reasonably expect a decrease of viscosity for necrotic cells [74]. However, the current model does not quantify the contribution of this effect into cell mobility and as a first approximation the mobility is taken constant for all cell phenotypes. The approximation of the total stress function *σ*_tot_(*σ*_*c*_, *σ*_ECM_) in the two-component media is based here on iso-strain model as most suitable for the description of transition layer (TL):

(3) $$\sigma_{\text{tot}}=\Phi_{c}\sigma_{\text{tot}}+\left(1-\Phi_{c}\right)\sigma_{\text{ECM}}$$

The Transition Layer can be described as spherical layer around external edge of MTS core, where the value of cell fraction Φ_C_ drops down significantly, forming the front of spheroid expansion in the radial direction out of MTS center. The total cell concentration on MTS front exhibits a sharp decrease from *C > C*_eq_ down to *C < C*_eq_. In other words, TL provides the transition from MTS core to invasive zone (IZ) represented by various forms of invasive patterns with low cell concentration compared to spheroid core. While the inner edge of TL is in the core, its outer edge is in IZ. As shown further in this work, TL plays significant role in MTS growth. This above description of MTS zones is purely phenomenological and based on data distributions obtained further in the current work, while no actual separation of MTS and its growing volume into zones or layers is assumed *a priori* by the current model.

#### 2.1.5 ECM permeability

One of the objectives of the present study is to verify assumption that the ECM stress built by the cells traveling through the scaffold, may change the transport conditions for these cells. This change may trigger a positive feedback loop in cell invasion when the invading cells may affect ECM structure in a way that stimulates their further invasion.

A substantially non-uniform stress distribution across the MTS edge has been reported in number of works [28], [29]. One of the key ECM characteristics influenced by the stress is the permeability of its scaffold. Permeability of collagens and other porous scaffolds reduces at compressive stresses [74] and is reasonably expected to increase when tensile forces are applied to the scaffold volume. The case of compression is well studied for diffusion of fluids through porous scaffold in tissue engineering and study biomechanics of biomaterials in Orthopedics [74], [75]. The data obtained by these researchers can be used as an indication of ECM permeability for cells, as function of stress. The mean pore size and consequently scaffold permeability *k* was shown to decrease with increasing compressive strain *ε* for Matrigel [76], as well as collagen-glycosaminoglycan (CG) scaffolds [74], so that *k* ∼ (1 − *ε*)^2^.

The permeability of stretched porous matrixes is not so well studied compared to the case of compression. However, a measurement on CG scaffolds demonstrates well expected result where cell migration increases with increasing pore size [77]. Also, a study of stretched porous membranes [78] showed a positive shift in the mean pore size and permeability that accompanies membrane stretching.

We can extrapolate the simple function presented in Ref. [74] for the compressive strain to the case of applied tensile strain assuming the mean pore size of the stressed matrix proportional to *l* (1 − *ε*)*.* Here the length *l* is a parameter of the porous microstructure and the strain *ε* is taken with negative sign for tensile case. Based on this simple approach, we can further describe the permeability of hydrogel porous matrix, undergoing tensile or compressive stress, as

$$k=k_{0}{\left(1-\frac{\epsilon_{v}}{3}\right)}^{2}\text{,}$$

where *ε*_*v*_ is ECM dilatation and *k*_0_ is permeability of unstressed hydrogel.

ECM undergoes symmetric tangential stretch and radial compression in front of the core spheroid, which represents the transition layer. As shown further in simulations, the total ECM stress distribution within TL ranges from tensile strain level up to maximum compressive values (Figures 6 and 7). This specific stress distribution may affect the conditions of tumor progression through ECM permeability.

For the case of stress-dependent ECM permeability the function (2) can be rewritten in the form:

(4) $$v_{D}=-M_{0}{\left(1-\frac{\epsilon_{v}}{3}\right)}^{2}\frac{\partial \sigma_{\text{tot}}}{\partial r}\text{,}$$

*M*_0_ is mobility of fluid in unstressed ECM *M*_0_ = *k*_0_/*μ*, where *μ* is the dynamic viscosity of the fluid.

### 2.2 Dynamics of cellular system

#### 2.2.1 Governing equations

We consider static linear elasticity problem for spherically symmetric system with the origin at the center of the sphere. The cells, moving due to their motility and also being under hydrostatic pressure are forced through hydrogel scaffold and proliferate in the pores, which causes expansion of the composite system. Similar process of solid tumor dilatation, conditioned by cell production and the reaction of cell–ECM composite system on stresses is described in models [73], [79].

ECM is considered here as solid elastic porous component. Its expansion results in spherically symmetric tensile stress and non-uniform stress distribution along the MTS radius.

These stresses, together with the intercellular stresses caused by cell–cell interactions, form the total stress distribution in cells–ECM composite in accordance with iso-strain model (3). Gradients of this stress yield hydrostatic conditions for cell motion according to (2), which leads to stress equilibrium in the system.

Here we reasonably assume that the growth rate of Glioma cells is rather slow compared to the viscous relaxation time in cell–ECM composite-quasi-static expansion. Therefore, the system is in stress equilibrium and its dynamics is provided by cell and oxygen balance equations.

The equation for the stress components:

$$\frac{d\sigma_{rr}}{dr}+\frac{1}{r}\left(2\sigma_{rr}-\sigma_{\theta \theta }-\sigma_{\phi \phi }\right)=0\text{,}$$

where *σ*_*rr*_ *, σ*_*θθ*_, *σ*_*φφ*_ are diagonal components of stress tensor.

Based further on strain-displacement and stress-strain relations, the governing equation for displacement *u* in the system [80], [81] can be written as

(5) $$\frac{\partial }{\partial r}\left(\frac{1}{r^{2}}\frac{\partial }{\partial r}\left(r^{2}u\right)\right)=\frac{\left(1+\nu \right)}{\left(1-\nu \right)}\delta_{v}\text{,}$$

where *ν* is ECM Poisson’s ratio; $\delta_{V}$ – the inner volumetric strain produced by expanding cell aggregation inside the matrix

$$\delta_{v}=\frac{1}{K_{\text{ECM}}}\frac{d\sigma_{C}}{dr}=\frac{\gamma K_{C0}}{K_{\text{ECM}}}\frac{d}{dr}\left(\frac{C_{\text{tot}}}{C_{\text{eq}}}\right)$$

*K*_ECM_ – ECM bulk modulus,

*K*_*C*0_ is the bulk modulus of the cell aggregation at equilibrium concentration *C*_tot_ *= C*_eq_

$$C_{\text{tot}}=C_{p}+C_{m}+C_{n}\text{;}$$

*C*_*p*_ – concentration of proliferating cells;

*C*_*m*_ – concentration of hypoxic cells;

*C*_*n*_ – concentration of necrotic cells.

Radial and tangential components of the strain tensor are as follows:

$$\epsilon_{rr}=du/dr;\epsilon_{\theta \theta }=\epsilon_{\varphi \varphi }=u/r$$

The components of ECM stress can be found as

(6) $$\sigma_{rr}=\frac{K_{\text{ECM}}}{\left(1+\nu \right)}\left(\left(1-\nu \right)\epsilon_{rr}+2\nu \epsilon_{\phi \phi }\right)\text{,}$$

(7) $$\sigma_{\phi \phi }=\frac{K_{\text{ECM}}}{\left(1+\nu \right)}\left(\nu \epsilon_{rr}+\epsilon_{\phi \phi }\right)\text{,}$$

as long as *σ*_*θθ*_ *= σ*_*φφ*_ in spherically symmetric system the total stress in ECM

(8) $$\sigma_{\text{ECM}}=\frac{2\sigma_{\phi \phi }+\sigma_{rr}}{3}\text{,}$$

where *σ*_ECM_ – total stress in ECM.

While the system is in quasi equilibrium of stresses its temporal dynamics is governed by cell and oxygen balance equations. Cell balance obeys reaction–diffusion model supplemented by advection, as cells drift in the hydrostatic force field. Equations (9–12) describe proliferation, invasion and transition of phenotype for each of the three cell types.

(9) $$\frac{\partial C_{i}}{\partial t}+\frac{1}{r^{2}}\frac{\partial }{\partial r}\left(r^{2}v_{D}C_{i}\right)=\frac{1}{r^{2}}\frac{\partial }{\partial r}\left[D_{i}r^{2}\frac{\partial }{\partial r}\left(C_{i}\right)\right]+S_{i}$$

where *C*_*i*_ is one of the three cell phenotypes *C*_*p*_, *C*_*m*_, *C*_*n*_, *D*_*i*_ are diffusion coefficients of *i*th type of cell: *D*_*p*_, *D*_*m*_ and obviously *D*_*n*_ = 0; *v*_*D*_ is drift velocity of cells, as defined by (2) for constant permeability of the scaffold and (4) for the case of stress-dependent ECM permeability. This velocity is the same for all types of cells in the model. *S*_*i*_ represents sources *S*_*p*_, *S*_*m*_ and *S*_*n*_ for proliferative, hypoxic and necrotic cells correspondingly.

(10) $$S_{p}=\frac{C_{p}}{\tau_{p}}\left(1-\frac{C_{\text{tot}}}{C_{q}}\right)\frac{n_{ox}}{n_{\text{max}}}-h_{pm}\frac{C_{p}}{\tau_{pm}}+h_{mp}\frac{C_{m}}{\tau_{mp}}\text{,}$$

(11) $$S_{m}=h_{m}\frac{C_{m}}{\tau_{m}}\left(1-\frac{C_{\text{tot}}}{C_{q}}\right)\frac{n_{ox}}{n_{\text{max}}}+h_{pm}\frac{C_{p}}{\tau_{pm}}-h_{mp}\frac{C_{m}}{\tau_{mp}}-h_{nec}\frac{C_{m}}{\tau_{n}}\text{,}$$

(12) $$S_{n}=h_{nec}\frac{C_{m}}{\tau_{n}}\text{,}$$

where *τ*_*p*_ and *τ*_*m*_ are characteristic times of proliferation for proliferative and hypoxic cells, *τ*_*pm*_ *, τ*_*mp*_ *, τ*_*n*_ are phenotype transition times: proliferative to hypoxic, hypoxic to proliferative and hypoxic to necrotic cells correspondingly. The maximum carrying capacity *C*_*q*_ is considered here as the upper cut-off limit assessed above as *C*_max_ = 8.3 × 10^5^ mm^−3^. The source of proliferative cells *S*_*p*_ represents logistic proliferation less transition of proliferative cells into hypoxic phenotype *C*_*p*_ *-*> *C*_*m*_. Reverse phenotype transition *C*_*m*_ *-*> *C*_*p*_, being rather slow process, also contributes into *S*_*p*_. The term *n*_*ox*_ */n*_max_ represents the ratio of oxygen concentration to its maximum level in the domain, which currently corresponds to the initial O_2_ concentration *n*_0_. This ratio approximates the decrease of proliferation rate at reduced concentration of oxygen; the approach was also used by other authors (e.g. [69]). The last two transition processes form hypoxic cell source *S*_*m*_ with the opposite sign along with the third term, which describes cell necrosis *C*_*m*_ *-*> *C*_*n*_. The fraction of necrotic cells is formed through necrosis of hypoxic cells only. The proliferative cells do not undergo direct transition to necrotic state. A deprivation of oxygen is the only condition resulting in cell death in the model, which goes through hypoxic stage.

The switch factors *h*_*pm*_ *, h*_*mp*_ and *h*_*nec*_ were introduced to provide a smooth approximation to the step function:

$$\begin{array}{c} h_{pm}\left(n_{ox}\right)=\left\{ \begin{array}{cc} 1, & \left(1-\tanh\left(n_{ox}-\xi_{h}\right)\lambda \right)/2\\ 0, & \left(1-\tanh\left(\xi_{h}-n_{ox}\right)\lambda \right)/2 \end{array}\right.\text{,}\\ h_{mp}\left(n_{ox}\right)=1-h_{pm}\left(n_{ox}\right)\text{,}\\ h_{nec}\left(n_{ox}\right)=\left\{ \begin{array}{cc} 1, & \left(1-\tanh\left(n_{ox}-\xi_{n}\right)\lambda \right)/2\\ 0, & \left(1-\tanh\left(\xi_{n}-n_{ox}\right)\lambda \right)/2 \end{array}\right.\text{,} \end{array}$$

where *ξ*_*h*_ and *ξ*_*n*_ are thresholds of oxygen concentration for *C*_*p*_ *-*> *C*_*m*_ and *C*_*m*_ *-*> *C*_*n*_ phenotype transitions correspondingly; *λ* = 1 provided smooth transition.

Factor *h*_*m*_ is defined according to one of the two approaches for cell phenotype transition exploited in the study. The first one assumes stiff “go or grow” approach, which means that proliferative cells exhibit no motility at all and hypoxic cells do not proliferate *h*_*m*_ = 0.

The soft “go or grow” approach has also been exploited assuming that all non-necrotic cells exhibit certain motility and mitosis. However, the rate of proliferation drops down for hypoxic cells, as well as rather low motility characterizes proliferative cells.

The oxygen concentration *n*_*ox*_ ≤ *ξ*_*n*_ is considered as complete deprivation of oxygen (and therefore threshold to anoxia) and defines the switch factor *h*_*nec*_.

Oxygen is critical metabolite and the only nutrient in this model. Diffusion of oxygen and its local consumption by the cells determine the local concentration *nox* as per equation (13).

(13) $$\frac{\partial n_{ox}}{\partial t}=\frac{1}{r^{2}}\frac{\partial }{\partial r}\left[D_{ox}r^{2}\frac{\partial }{\partial r}\left(n_{ox}\right)\right]-\left(\alpha_{p}C_{p}+\alpha_{m}C_{m}\right)$$

*n*_*ox*_ is oxygen concentration, *D*_*ox*_ – diffusion coefficient of oxygen, *α*_*p*_ and *α*_*m*_ – oxygen uptake for proliferative and hypoxic cells correspondingly.

Following (3) we can write for the total stress in the composite system:

(14) $$\sigma_{\text{tot}}=\left(\frac{C_{\text{max}}-C_{\text{tot}}}{C_{\text{max}}}\sigma_{\text{ECM}}+\frac{C_{\text{tot}}}{C_{\text{max}}}\sigma_{C}\right)\text{.}$$

#### 2.2.2 Initial and boundary conditions for the governing equations

Following Stein’s et al. experimental setup [68] the initial radius of the spheroid in simulations has been set to *R*_0_ = 250 μm and cell concentration *C*_init_ = 7.0 × 10^4^ cells/mm^3^.

We prescribe a uniform initial distribution of oxygen in the domain *n*_*ox*_(*r*, *t* = 0) = *n*_0_ = 4 mg/L.

Spherical symmetry dictates symmetry conditions for all profiles *X*_*i*_ about *r = *0: *dX*_*i*_/*dr* (*r* = 0) = 0*.*

We assume zero displacement and strain on the distant border of the domain *r = R*_0_:

$$\begin{array}{c} U\left(r=R_{0}\right)=0\\ \epsilon_{rr}\left(r=R_{0}\right)=0 \end{array}$$

To prevent cells from leaving the domain no-flux boundary conditions were imposed for all cell phenotypes in equation (9): *dC*_*i*_/*dr* (*r = R*_0_) = 0*.*

Two types of BCs were considered for oxygen diffusion (13).

**Type 1:**

Closed volume or reflection boundary conditions (RBC)

This assumes the same condition on the domain border as set for cells

$$dn_{ox}/dr\left(r=R_{0}\right)=0\text{.}$$

**Type 2:**

Permanent supply (PSBC)

This condition assumes permanent supply of oxygen throw the border, so that

$$n_{ox}=n_{0}=\text{const}.$$

#### 2.2.3 Numerical implementation

Finite differences approximation has been used to solve the system (5)–(14). Equation (5) is discretized by a second order symmetric scheme. Crank–Nicolson scheme was implemented to approximate solution to the oxygen and cell diffusion problems (9) and (13). Time splitting approach is used to approximate phenotype transition and cell transport due to stress gradients. There was special requirement for conservative and stable algorithm to approximate the problem of hydrostatically forced cell drift in the condition when the force-field changes its sign along the MTS radius. The Phoenical LPE version of Flux Corrected Transport (FCT SHASTA) algorithm has been implemented to solve this problem [82]. This symmetric three point diffusive transport scheme provided second order of approximation, which is important to form a correct cell density profile of the invasive zone and also stable solution in stressed MTS core.

### 2.3 Choice of parameters

#### 2.3.1 Mobility of glioma cells in collagen

Mobility is calculated as the value of collagen permeability ***k*** over dynamic viscosity of cells *μ*: *M = k*/*μ*. The results obtained for cultured anaplastic carcinoma cells [83] were taken as a reference value of cancer cells viscosity 300 Pa s.

There are dramatic variations in the experimental data for collagen permeability ranging from 2 × 10^−16^ m^2^ for 3% collagen [84] to 1.00 × 10^−12^ m^2^ [85]. Other authors typically show data within the above range, for example 10^−15^−10^−14^ m^2^ [86], [87]. Taking the top value of permeability 1.00 × 10^−12^ m^2^ we can obtain *M* = 3.3 × 10^−15^ m^2^/(Pa s) = 2.9 × 10^−4^ mm^2^/(Pa d). This choice of permeability value brought the results of simulation into correspondence with the experimental data.

#### 2.3.2 Rheological parameters

Similar to permeability values the variations in the stiffness of collagens used for culturing GBM cells are also significant. Typically, GBM cells are being cultured in collagens of *E* = 1.0 and 35 kPa [6], [17], [88]. Some measurements show much lower values. For example, the examination of gel in the absence of cells used further for Glioma MTS experiments [15] showed Young’s moduli of *E* **∼** 100 Pa at concentrations 1.5–2.5 mg/mL. The study of acellular collagens in Ref. [89] gave typical range for Young’s modulus *E* = 0.5–12 kPa with Poisson ratio *ν* ∼ 0.13. In particular, acellular crosslinked gels of 3–5 mg/mL exhibited stiffness of 1–2 kPa. These detailed results were taken as a basis for our calculations. The range *E* = 0.5–2.5 kPa has been studied, which gives for bulk modulus *K* = 225–1130 Pa at *ν* = 0.13.

As discussed above, a cell colony represents compressible media. Compared to hydrogels, cancer cell aggregation exhibit lower compressibility with Poisson ratios about *ν*_*C*_ = 0.36–0.4 [63], [90].

Rheological parameters of porous cell aggregation without any other inclusions should ideally represent cells in the model as a component of the general composite system. Our evaluation of these parameters was based on stiffness of the Glial Cells *E*_cell_ ∼ 200–300 Pa [91] and Young’s moduli derived from the surface of fresh glioma samples 350 Pa [88]. We take *ν*_*C*_ = 0.4; *E* = 300 Pa and *K* = 500 Pa.

#### 2.3.3 Cell diffusion in collagen

The diffusion coefficients for the model *D*_*h*_ are determined by the random motility of invasive hypoxic cells. The sources are being mostly referred to in literature show invasive diffusivity for hypoxic cells from *D*_*h*_ *= *1.0 × 10^−3^ mm^2^/day [92] to *D*_*h*_ = 2.0 × 10^−2^ mm^2^/day [93].

In glioma MTS experiments of Stein et al. [68] these data were validated through comparison with the measured characteristics. It was found that the value of *D*_*h*_ = 2.0 × 10^−2^ mm^2^/day most closely described the measured MTS growth speed. This particular value was used in the current model along with typical approach for normal proliferative cells *D*_*p*_ = *D*_*h*_/10 [47]. The comparison of the results of simulation based on this particular couple of diffusion coefficients *D*_*p*_, *D*_*h*_ provided rather good agreement not only with MTS growth characteristics obtained in Ref. [68], but also described well the experimental density distribution on the MTS front.

#### 2.3.4 Oxygen transport and consumption

**Diffusion** coefficient *D*_O2_ varies in sources within the range *D*_O2_ = 86.4–160 mm^2^/day [94], [66]. In the current work the range of its variation *D*_O2_ = 40.0–150 mm^2^/day has been studied and the value of *D*_O2_ = 86.4 mm^2^/day provided the best fit to the experimental data of Ref. [68].

**Oxygen uptake *α***_***p***_ **for *C***_***p***_ varies insignificantly 1.38–2.8 × 10^−2^ (mm^3^/cell) mg/(L*day) [47], [66]. The value ***α***_***p***_ **= **1.4 × 10^−2^ (mm^3^/cell) mg/(L*day) has been used for the calculations, as it keeps proliferation rate close to experimentally observed values.

**Oxygen uptake for *C***_***h***_ ***α***_***h***_ **= *α***_***p***_ **/5** was taken as typical approach [47], [95].

#### 2.3.5 Proliferation and phenotype transition

**Proliferation Rates** vary, but insignificantly and typically for *C*_*p*_ the doubling time is ***τ***_***p***_ = 1.0 day while for hypoxic cells ***τ***_***h***_ = 2.0 day [41], [47], [96].

**Phenotype switch time** (proliferative to hypoxic) *τ*_*ph*_ *= *1 h, (hypoxic to proliferative) *τ*_*hp*_ = 96 h [47]. Phenotype switch time (hypoxic to necrotic) 3.1 × 10^−2^ h^−1^ = 0.74 day^−1^ [52]

**Switch thresholds *C***_***ph***_ **and *C***_***hn***_

In spite of dramatically large scale of data variation for phenotype transition thresholds [97], [98], [99] their actual values did not have noticeable effect on the process dynamics. The basic threshold values of oxygen concentration used in simulations (Table 3) have been chosen simply as mostly used by other authors. The further calculations show that variations of thresholds influences mostly initial stage of spheroid growth, but has rather moderate effect on total cell profiles of 3–5 days grown MTS.

## 3 Calculation results

In order to study the process sensitivity to the model variation, some model elements and parameters have been varied around the basic model configuration. The basic approach includes the overlapping cell phenotype switch and assumption of constant ECM permeability, as per (2). The basic assumption for oxygen supply is closed domain, which means that no oxygen is being supplied through the domain border and corresponds to reflection boundary conditions (RBC). Another condition for permanent oxygen supply (PSBC) is applied in calculations for comparison purposes only. The influence of variable permeability, as a function of strain according to (4), has been studied as variation of the basic model. Also, the stiff phenotype switch approach that strictly follows GoG rule has been studied as a variation of GoG model. The major calculations and comparison with experimental data is carried out in the frame of the basic approach. The comparative analysis of the variations is presented with regard to the results of the basic model.

### 3.1 Formation of cell density profiles

The results obtained in calculation are generally in line with the experimental data of Stein and colleagues [68] for two cell lines U87WT and U87ΔEGFR. Based on the observed cell density distribution the authors identified two main MTS zones. These were the bulk part of the spheroid-MTS Core and the Invasive Zone – the peripheral area of the growing spheroid where cell density drops from typical core values down to zero. There were threshold cell densities introduced in Ref. [68] to define the separation border between the core and IZ. The outer boundary of IZ is defined as a cut off cell density level on the outer edge of the Invasive Zone. These levels were different for the two studied cell lines: *C*_IZ1_ = 10^4^ cells/mm^3^ and *C*_IZ2_ = 2.5 × 10^3^ cells/mm^3^ for U87WT and U87ΔEGFR correspondingly. The border between the core part and IZ was formally defined in Ref. [68] as a rim with cell densities *C*_CR1_ ≈ 10^5^ cells/mm^3^ and *C*_CR2_ ≈ 8 × 10^4^ cells/mm^3^ for the two studied cell lines.

The cores and IZs of the U87WT and U87ΔEGFR spheroids exhibit different invasiveness as well as different characteristic cell densities [68]. The current model based on average mechanobiological properties of glioma cells does not take into account the difference in individual characteristics of the two cell lines. Once the model is unable to quantify this difference we can speak here only about qualitative comparison of the simulations with the experimental results. Not carrying out a fitting to the data of any particular one of the two cell-lines studied in the experiment we present here simulation results obtained for the average parameters taken for glioma cells, according to Tables 1, 2, 3 and 4.

**Figure 1:**
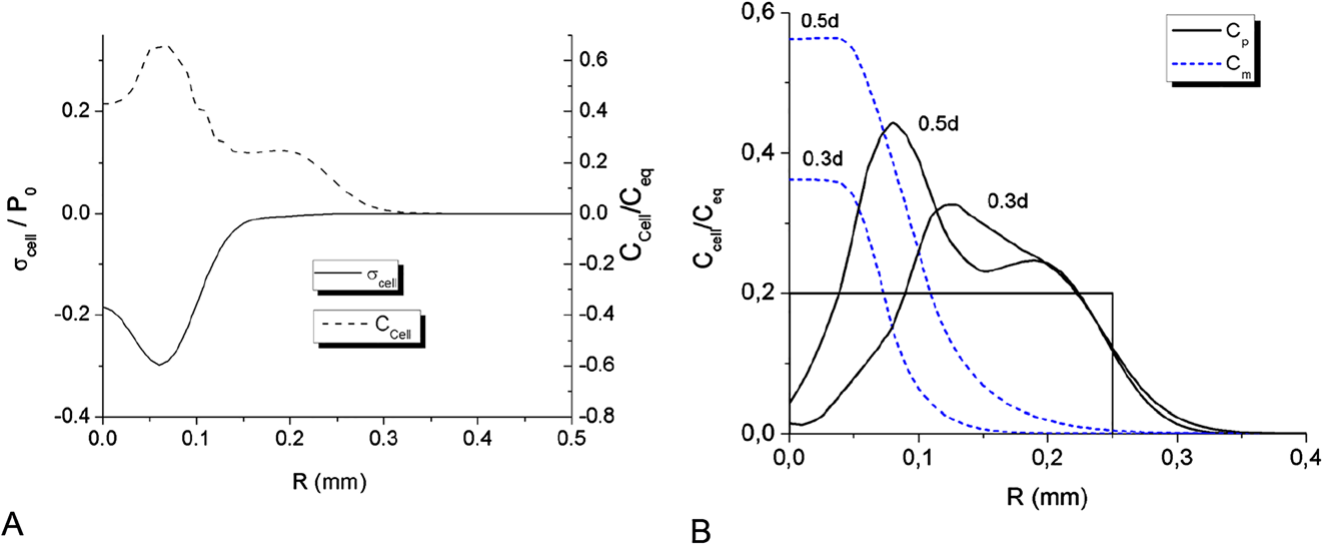
(A) Radial distribution of cell–cell traction forces *σ*_Cell_/*P*_0_ and cell concentrations *C*_Cell_/*C*_eq_ for *t* = 0.3 day; ***C***_***eq***_ = 3.5 × 10^5^ cells/mm^3^, *P*_0_ = 100 Pa. (B) Proliferative (solid lines) and hypoxic (short-dashed lines) cell density distributions for the moments of time *t* = 0, 0.3 and 0.5 day. initial distribution (*t* = 0) represents proliferative cells only-rectangular area on the graph.

As the initial cell density is well below equilibrium value *C*_init_ = *C*_eq_/5, cell–cell adhesion represents dominating intercellular force in the whole spheroid. The distribution of cell–cell traction forces is presented on Figure 1A for *t* = 0.3 day. The intercellular stress curve exhibits non-monotonic tensile character, which is determined by local cell concentrations and *F*–*D* function for glioma cells, according to (1a).

**Figure 2:**
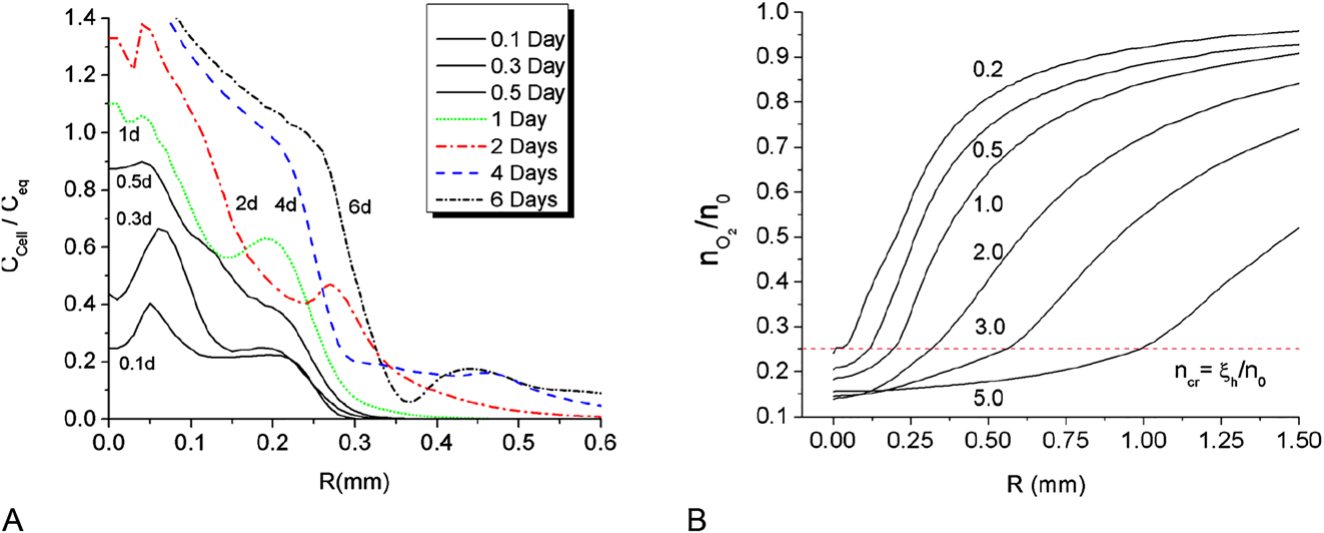
(A) Distributions of total cell densities for *t* = 0.2–5.0 days; (B) distribution of oxygen concentrations for *t* = 0.2–5.0 days; The line *n*_*cr*_ *= ξ*_*h*_/*n*_0_ shows hypoxic transition threshold level; *n*_0_ is initial oxygen concentration, *n*_0_ = **4** mg/L..

In the beginning of MTS growth the inner traction forces cause contraction of MTS core part. Figures 1B and 2A illustrate this process as formation of high density spherical layer and its shift towards center, which ultimately leads to densification of MTS center within the first 12 h. The initial contraction was also observed in Stein’s et al. experiment as slight decrease of radius of the core spheroid.

Two processes are forming the distribution of cell density immediately after the initial spheroid is placed into the hydrogel: spheroid contraction and separation of initially proliferative cells into different phenotype groups.

**Figure 3:**
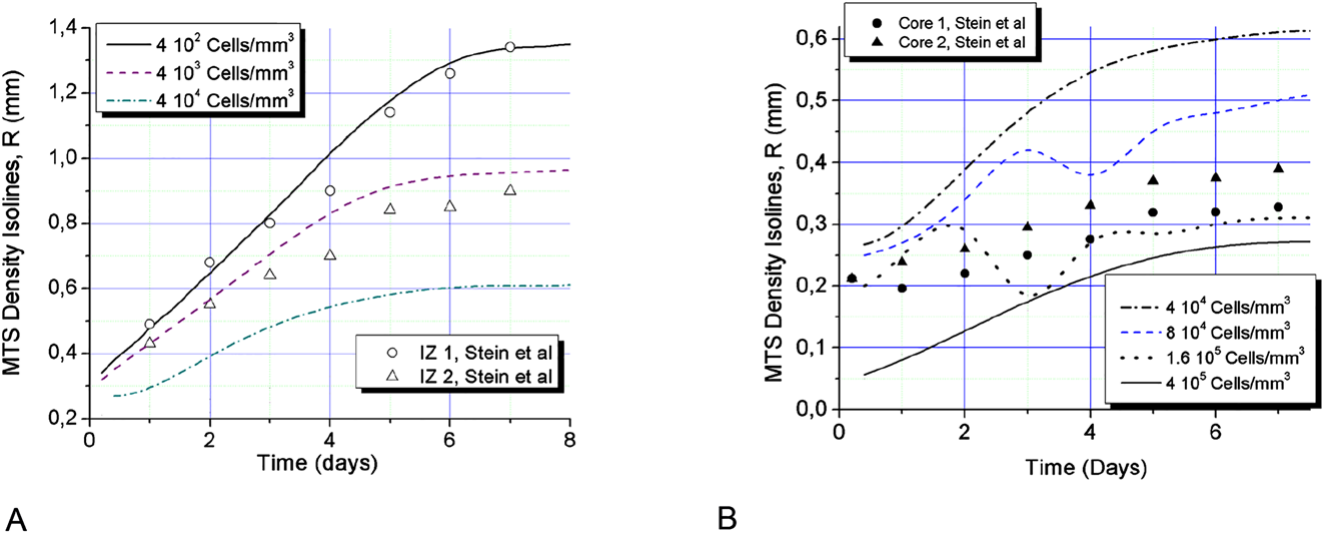
Isolines of cell concentrations obtained in simulation of the experimental conditions of Stein’s et al. [68]; (A) Solid black line corresponds to cell concentration of 400 cells/mm^3^, dashed line – 4 × 10^3^ cells/mm^3^, dashed-dotted line is for 4 × 10^4^ cells/mm^3^ and presents on both graphs (A) and (B); open circles show experimental data obtained by Stein et al. for U87WT glioma cell line (*C*_IZ1_ = 10^4^ cells/mm^3^), open triangles – the results for U87ΔEGFR cells (*C*_IZ2_ = 2.5 10^3^ cells/mm^3^); (B) solid black line denotes equilibrium cell density in the model *C*_eq_ = 4 × 10^5^ cells/mm^3^, dotted line corresponds to the concentration of 1.6 × 10^5^ cells/mm^3^, blue dashed – 8 × 10^4^ cells/mm^3^ and dash-dotted – 4 × 10^4^ cells/mm^3^; circles and triangles show the core boundary isolines *C*_CR1_ ≈ 10^5^ cells/mm^3^ and *C*_CR2_ ≈ 8 × 10^4^ cells/mm^3^ obtained in experiment for the two above mentioned glioma cell lines correspondingly.

The hypoxic condition, being a triggering factor of phenotype transition in the model is achieved in the spheroid center during the first 8 h of its growth, when oxygen concentration drops below critical level *ξ*_*h*_ = 1.0 mg/L (Figure 2B). Time-limited diffusion of oxygen to the center of spheroid forms specific radial distributions of its concentration at different moments of time, which is one of the major factors determining radial cell density profile (Figure 1B and 2A). The oxygen profile also determines partial concentrations of different cell phenotypes in the total cell population. Figure 1B shows growing accumulation of hypoxic cells in the center and initial expansion of proliferative zone for 7.2 and 12 h. Once the phenotype transition began, the cell diffusion out of the core spheroid, which models cell motility, is growing up quickly and forms low density protrusion (Figure 3A). However, cell motility does not affect MTS expansion at its early stage, as the nearest external layer of the core spheroid is formed dominantly by proliferative cells and expands due to cell proliferation in the area of relatively high oxygen concentration in front of this layer. The phase velocity of proliferative layer expansion (Figure 3B) agrees well with *in vitro* Stein’s et al. data. The solid line on Figure 3B represents a rim of equilibrium cell density in the simulated MTS core where the intercellular traction is compensated by the repulsion forces. Above this isoline, the average intercellular distances exceed the equilibrium value, therefore traction forces dominate.

**Figure 4:**
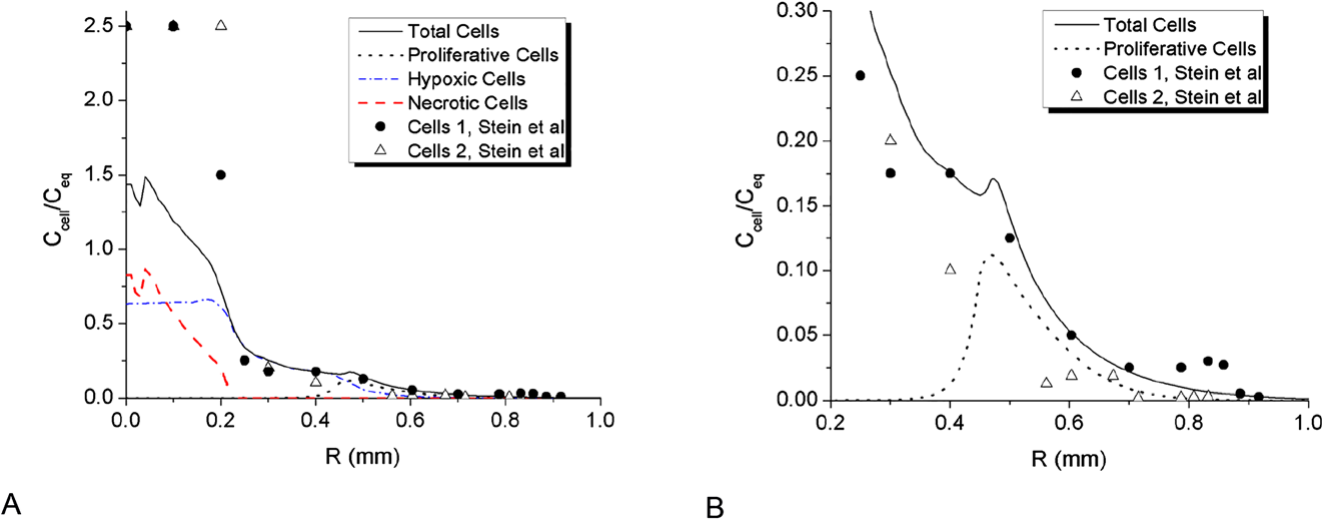
Comparison the results of calculation with experimental data for day 3 of MTS growth obtained in [68] for the U87WT cell line (solid spheres). The figures present calculated distributions of total cell density obtained for *D*_O2_ = 86.4 mm^2^/day; Proliferative, hypoxic and necrotic cells in different scales for clear comparison (A) and (B).

**Figure 5:**
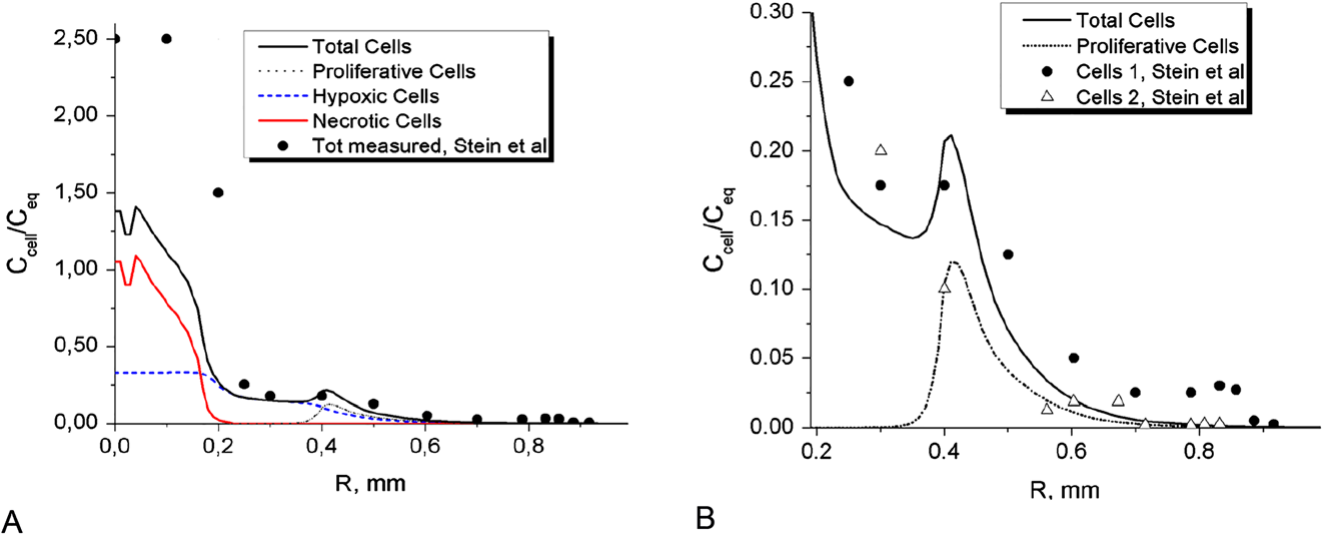
Comparison the results of calculation with experimental data for day 3 of MTS growth obtained in [68] for the U87WT cell line (solid spheres). The figures present calculated distributions of total cell density obtained for *D*_O2_ = 40.0 mm^2^/day; Proliferative, hypoxic and necrotic cells in different scales for clear comparison (A) and (B).

The initial cell motion towards the spheroid center slows down while the central density grows up to *C*_eq_. At the same time, the invasion of motile cells into the surrounding porous scaffold out of MTS core forms extended IZ within the first two days, as shown on Figure 2A and further on Figures 3, 4 and 5. These two processes: spheroid contraction with densification of its center and the fast development of low density IZ provide substantial redistribution of MTS density during the first day of its growth (Figures 1B and 2A). At further MTS growth, this elongated shape of cell density profile across its border does not change significantly.

The results of the simulation satisfy well the measured characteristic times of the core development, which exhibits rapid expansion and further saturation. IZ expansion curves also show rapid initial growth of IZ over 200 μm within the first day (Figure 3). Though, the cell densities do not correspond exactly to the levels measured in experiment.

The expansion of IZ saturates within five days (Figure 3A) and the growth of the MTS core also shows signs of saturation (Figure 3B), which is also in line with the experimental results. The two isolines of cell densities 4 × 10^2^ and 4 × 10^3^ cells/mm^3^ (Figure 3A) correspond well to time-dynamics of IZ borders observed by Stein and colleagues [68]. The distribution of cell density along the MTS radius is generally non-monotonic, see Figures 1A and 2B.

Non-monotonic growth character can be also seen on cell density isolines (Figure 3B) in the density range 8 × 10^4^–1.6 × 10^5^ cells/mm^3^. This is the range of strongest cell–cell adhesion and therefore intercellular traction forces. The traction forces build local cell agglomerations in IZ that grow fast at relatively high oxygen content in front of the spheroid. Similar local steps and elevations in the invasive zone of tumor can be seen on cell density profiles obtained in experiments (Figures 4 and 5). At certain conditions these agglomerations may grow fast suppressing cell growth around them through consumption of locally available oxygen and forming long protrusions and subtumors. These effects are considered further in this work.

As calculations demonstrated (Figures 4 and 5), cell density profiles are rather sensitive to oxygen diffusion, which does not fully replenish its consumed volumes inside MTS. The diffusion of oxygen determines to great extent the intensity of phenotype transition along the MTS radius and therefore cell density profile. The simulation demonstrates typical dynamics of MTS growth when the proliferative cells quickly turn into hypoxic phenotype in the central area (Figure 1B) and by day 3 the central part is mostly necrotic (Figure 4A). The proliferative cells are confined within a thin layer in the outer part of MTS (Figure 4B at *R* ≈ 0.48 mm and Figure 5B at *R* ≈ 0.43 mm). The figure also shows sharp vertical edge of cell density profile observed in experiment and tenfold rise of cell concentration in the center compared to its initial value. The rise cannot be explained by local cell proliferation only. This is presumably a result of compression from expanded ECM scaffold and cell–cell adhesion effect in the initial spheroid. The effect of spheroid contraction is also seen on simulated profiles, but it is not that strong as in experiment. The simulation data shows about sevenfold increase of cells density in the center. There are set of parameters responsible for this rise, such as ECM stiffness and permeability, diffusion of oxygen and barriers of cell phenotype transition. A detailed analysis of their combined effect on maximum cell density has not been carried out. However, a variation of any single parameter did not provide better agreement with the experimental results. The simulated cell density in the very center was always only 60% of that obtained in experiment.

The sensitivity of MTS growth to oxygen diffusion coefficient is illustrated by Figure 5. The diffusion coefficient has been reduced down to *D*_O2_ = 40.0 mm^2^/day, which is less than a half of that reported in literature, see Table 3. This relatively low diffusion coefficient sharpens the edge of the core spheroid (Figure 5A) and slows down its growth. This moderate effect of oxygen diffusion on the cell density profile is well expected and predictable.

Generally, the rise of total cell density in the middle of IZ obtained in simulation is conditioned by thin proliferative rim (Figures 4B and 5B) located outside of the core spheroid at *R* ≈ 0.43–0.48 mm. The profile measured in experiment for U87WT cell line also exhibits a clear step around the same point of *R* ≈ 0.43 mm, which is presumably a sign of proliferative rim.

The measured by Stein et al. density distribution of three days MTS for U87WT cells shows another rise of cell density at the end of IZ at *R* ≈ 0.85 mm, which was not exactly reproduced in simulation.

The simulated Stein’s conditions generally result in non-monotonic radial profiles of cell concentrations (Figure 2A). This situation may theoretically lead to formation of distant cells clusters in the form of additional proliferating rims. Similar scenario was identified by other researchers as formation of subtumors [55]. Cell–cell adhesion–repulsion has been reported as major mechanism responsible for this effect. We also carried out simulation of subtumors and consider conditions for their formation further in this work.

Though the calculated core densities are generally lower in simulation, the comparison with Stein’s et al. data demonstrates qualitative agreement. The calculated cell density profiles exhibit similar dense core with a steep density fall at its edge and elongated IZ presenting a shelf with steps or small distant rise of cell density (Figures 4 and 5). In so doing, the comparison shows better agreement with the data obtained for U87WT cell line. This cell line exhibits relatively smooth IZ, which should therefore closer correspond to our 1D simulation of radial MTS growth than clearly discrete protrusions in IZ obtained by Stein for the another cell line U87ΔEGFR.

It should be noted that all calculations carried out for comparison with the experimental data have been prepared at RBC, as a basic case. These boundary conditions assume closed domain with limited total oxygen content inside it. MTS growth at RBC inevitably saturates and the saturation time is obviously monotonic function of the domain volume. In so doing, the main proliferation rim exhausts completely. In the absence of proliferative cells MTS expansion slows down considerably, while the growth of IZ is observed within 1–2 extra days width decreasing speed (Figure 3). This leads to further elongation of cell density profile, which produces an effect of its slow expansion.

In case of permanent supply of oxygen through the domain border (PSBC case) the proliferative layer is more stable and oxygen consumption is limited by its diffusion only. This case has also been simulated in the current work.

### 3.2 The effect of stress distribution and variable permeability

**Figure 6:**
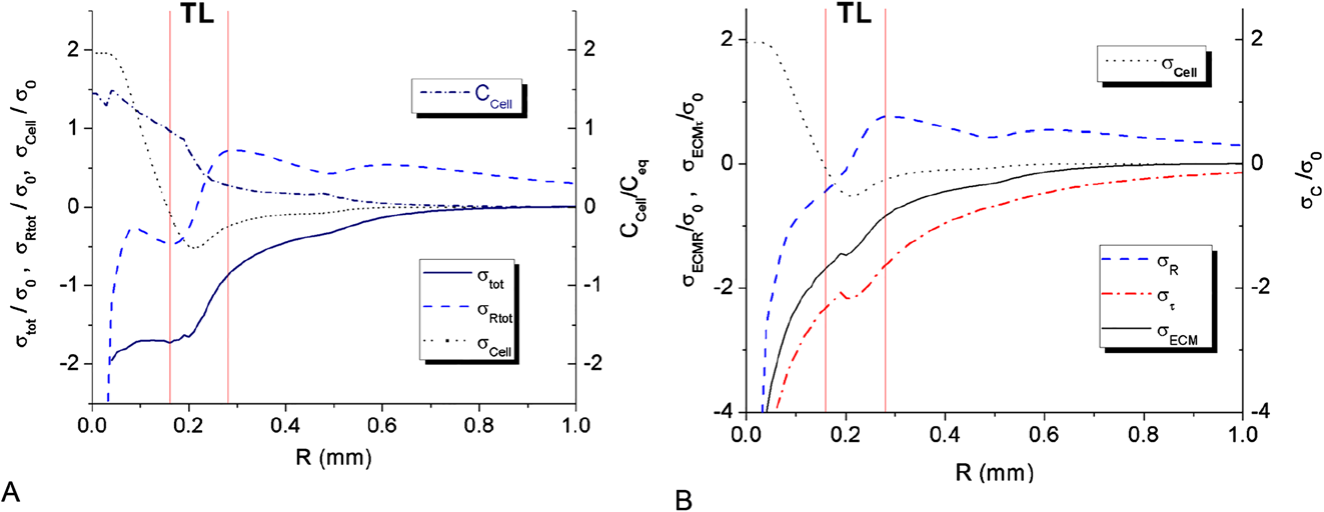
Stress distribution along MTS radius at day 3. (A) The total stress distribution *σ*_tot_/*σ*_0_ is calculated within iso-strain model; the graph also shows radial component of the total stress *σ*_Rtot_/*σ*_0_, the stress conditioned by cells interaction *σ*_Cell_/*P*_0_ and concentration of cells *C*_cell_/*C*_eq_, *σ*_0_ = 100 Ps; (B) distribution of ECM stress *σ*_ECM_/*σ*_0_, tangential component of ECM stress *σ*_*τ*_/*σ*_0_ and its radial component *σ*_*R*_/*σ*_0_.

**Figure 7:**
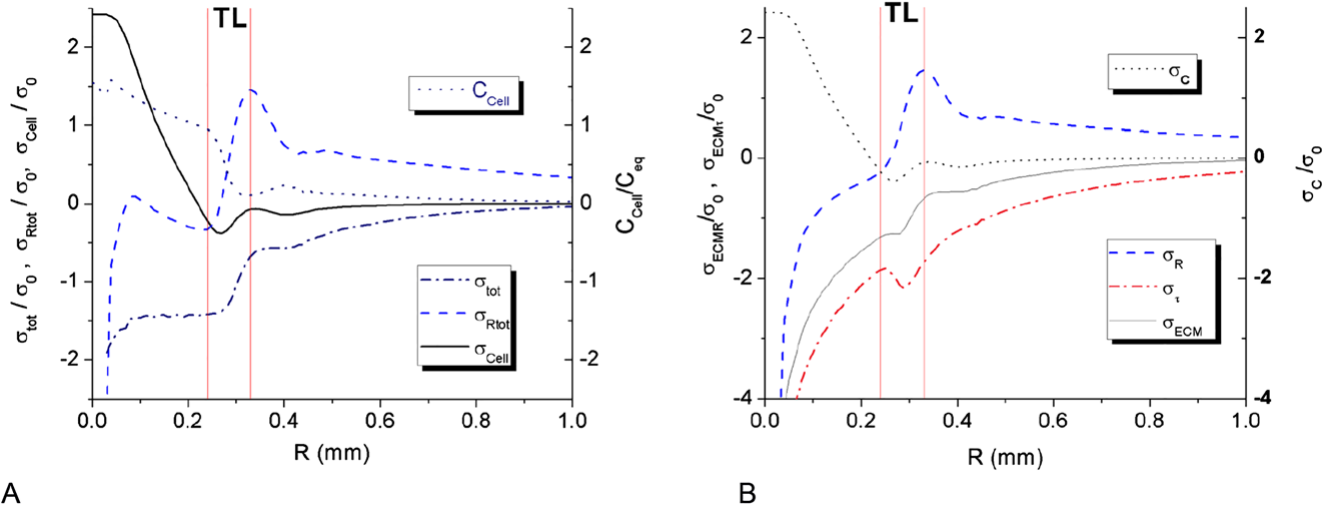
Stress distribution along MTS radius at day 5. (A) The total stress distribution *σ*_tot_/*σ*_0_ is calculated within iso-strain model; the graph also shows radial component of the total stress *σ*_Rtot_/*σ*_*0*_, the stress conditioned by cells interaction *σ*_Cell_/*σ*_0_ and concentration of cells *C*_cell_/*C*_eq_, *σ*_0_ = 100 Ps; (B) distribution of ECM stress *σ*_ECM_/*σ*_0_, tangential component of ECM stress *σ*_*τ*_/*σ*_0_ and its radial component *σ*_*R*_/*σ*_0_.

The expansion of MTS resulting from cell proliferation and their intrusion into the surrounding porous scaffold creates significant tangential tension (Figures 6 and 7). The tension value exhibits a sharp raise around the spheroid center. The calculated values of ECM tension are based on the assumption of pure hydrogel elasticity, which works well enough in the whole domain except for the very center of the spheroid. The raise of tension in MTS center is obviously limited by hydrogel yield point and should lead at this point to partial stress relaxation in the center. In the model we do not consider ECM plasticity simply keeping the high values of stress in the spheroid center. In calculation this high ECM stress in the middle does not have any noticeable effect on MTS dynamics, as the partial concentration of ECM fraction is rapidly dropping down towards the spheroid center.

In the outward direction along the spheroid radius the radial component of total stress in the cell-matrix composite exhibits a sharp transition from tensile to compressive stress (Figures 6A and 7A). This transition is conditioned to a great extent by ECM reaction on the expansion of MTS, forming similar stress distribution in the matrix within relatively thin near-MTS-surface layer (Figures 6B and 7B). The pressure exerted on the matrix from the expanding MTS creates this transition layer (TL), as described above, with relatively high radial compression of ECM and tangential tension at the same time (Figures 6B and 7B).

The expansion of cell aggregation behind TL causes both radial and tangential stretching of the extracellular scaffold in the bulk spheroid (Figures 6B and 7B). The total stress in the system is tensile and exhibits nearly constant value inside the core, see the negative level shelf on Figures 6A and 7A and a sharp step up within the TL. In general, even simple model of a single elastic incompressible continuum [33] shows a tension zone in the center of expanding tumor spheroid and radial compression on its periphery. But that simple approach does not produce the fine structure of stress distributions.

The constant level of the total stress in the composite system is being formed through mechanical interaction between its two constituents – cells and ECM. The hydraulic pressure built by proliferating cells is decreasing along the MTS radius following the decrease of total cell concentration, which is still above *C*_eq_ and therefore causes compressive stress inside the spheroid behind TL. In the transition layer on spheroid surface the cell concentration drops below *C*_eq_ creating certain tensile forces in cell aggregation due to dominating intercellular adhesion (Figures 6A and 7A). The positive intercellular pressure produces compensatory effect against tensile stress in the ECM scaffold inside the spheroid. This effect is illustrated by two curves *σ*_*C*_ (*R*) and *σ*_ECM_ (*R*) demonstrating opposite sign stresses within the two constituents of the composite system (Figures 6B and 7B). As a result of this effect the total stress calculated within the isostrain composite model is nearly constant throughout major part of the spheroid *σ*_tot_ (Figure 7A). This constant value of stress inside MTS is being maintained by self-consistent process that involves interaction between cells and ECM, resulting in redistribution of stresses and cellular drift. This drift modulates the distribution of cell concentration in accordance with the total stress gradients inside the spheroid.

The sharp raise of the total stress across the transition layer builds hydrostatic pressure gradients within this narrow area directed outwards across the TL. This layer represents a barrier for diffusive cell transport out of MTS core. The resulting pressure force steepens the front of cell density distribution, pushing back some of the motile cells traveling out of the core. Nevertheless, as simulations demonstrate, the highly motile cells typically overcome this pressure barrier in cases of soft and moderately rigid ECMs.

Apart from the particular effect of stress on ECM pore size, the matrix structure and its mechanical reordering are not simulated in the current work. The effect of stress on ECM structure is considered here in the frame of simple model and may depend on the matrix type.

The specific stress distribution within TL obtained in simulation may presumably affect the structure of ECM scaffold. In case of randomly arrayed fibers they should expectedly be rearranging in front of TL, taking directions normal to MTS radius. The fibers should undergo further reorientation within TL along spheroid radius, as radial stress components turn tensile inside the spheroid. This scenario of mechanical remodeling of collagen scaffold has been observed *in vitro* [29] on murine colon carcinoma. The authors have shown the tangential alignment of the collagen fibers around the spheroid surface and general radial tension inside it. These general traction forces (Figures 6A and 7A) are assumed to facilitate tumor invasion providing tracks through mechanical alignment in matrix structure [28], [29]. The ability of collagens to remodel and in particular to align under external strain forces applied has been demonstrated in experiments [100] as a typical property of fibrous biological networks. This effect of mechanical remodeling, in the frame of the current model should technically result in local increase of ECM permeability. However, the observed phenomenology is still not studied well enough to quantify its influence within the model. We can only note that the stress distribution obtained in simulation may practically condition the observed experimentally mechanical restructure in ECM.

It must be stressed that there is a principal difference between the current two-constituents’ composite model and a single-constituent only cellular models. In single constituent models, where only hydrostatic pressure inside the proliferating cell colony determines its expansion [68], [69], [73], the elastic reaction of extracellular constituent on this expansion is often ignored. A regime of stationary MTS growth assumes no intercellular pressure *σ*_*c*_ ≈ 0, keeping cell concentrations around equilibrium value *C*_eq_ within a single constituent self-consistent model [69].

In contrast to these simple cases the present model takes into account the mutual interaction of the two compressible constituents of the composite system: visco-elastic cell aggregation and elastic matrix within the whole simulation domain. This interaction forms a specific cell density profile inside the spheroid and a thin transition layer on its surface, where most mechanical characteristics of the composite system undergo rapid transition.

### 3.3 Formation of subtumors

**Figure 8:**
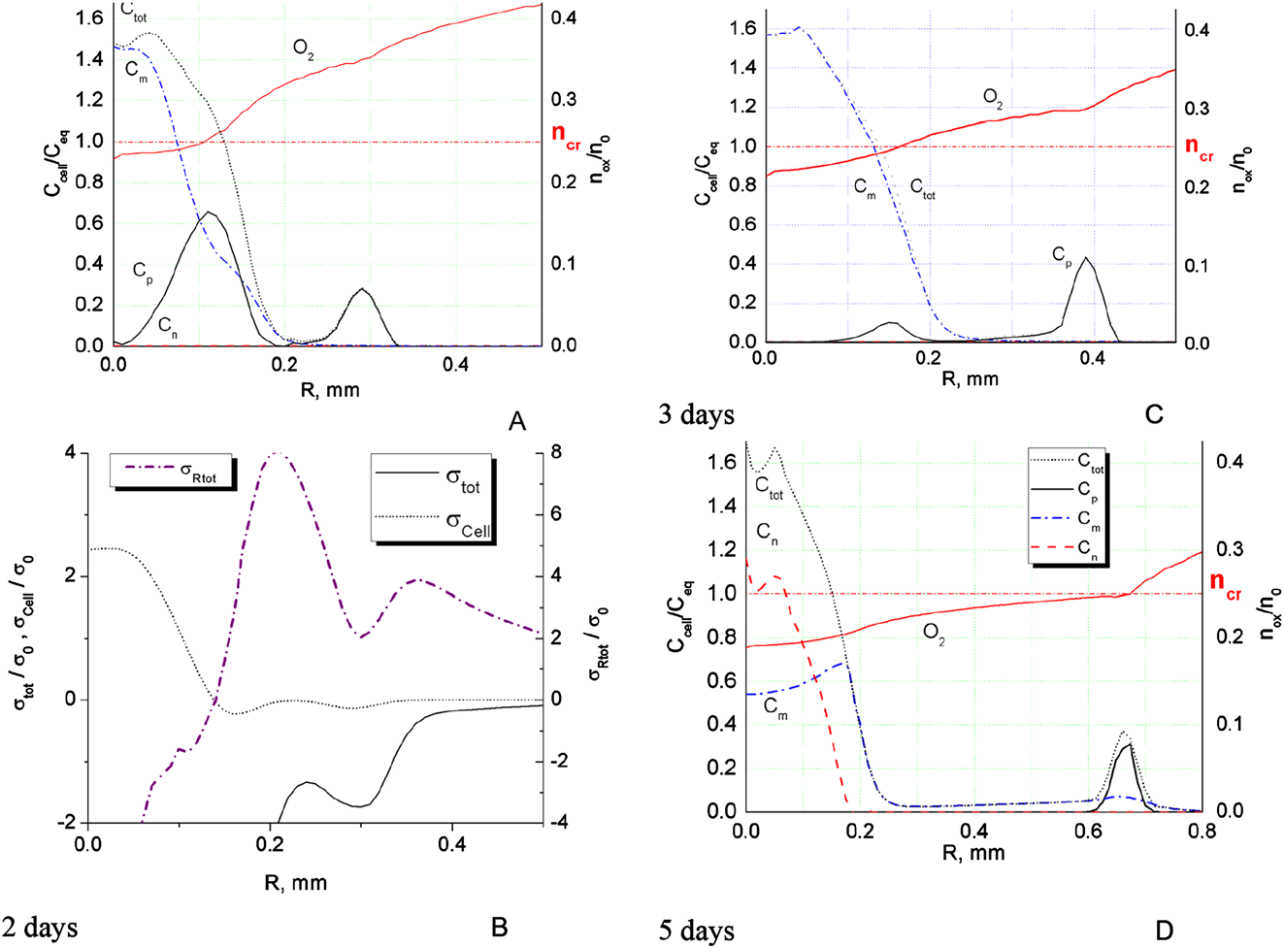
Formation of subtumor. Calculation results presented for days 2, 3, 5 of MTS growth at elevated matrix stiffness *E*_ECM_ = 2.5 kPa and PSBC. The graphs show radial cell density distributions (A, C, D): for proliferative cells *C*_*p*_, hypoxic *C*_*m*_, necrotic *C*_*n*_ and total cell density *C*_tot_; also the distribution of oxygen concentration is shown on these graphs; the dashed line *n*_*cr*_ *= ξ*_*h*_/*n_0_* shows the threshold level of cell phenotype transition proliferative-to-hypoxic, *n*_0_ = 4 mg/L; (B) shows stress distribution for day 2 including total stress in cells–ECM composite *σ*_tot_, intercellular stress distribution *σ*_cell_ and radial component of the total stress *σ*_Rtot_.

The next series of calculations has been carried out at high ECM stiffness *E*_ECM_ = 2.5 kPa and permanent oxygen supply through the domain border (PSBC). These specific parameters conditioned formation of subtumor. There are no laboratory data for comparison and the simulations have been carried out in order to demonstrate this effect as potential scenario discussed earlier in Refs. [55], [56], [57]. The matrix stiffness is selected so that provides substantial contraction of the initial spheroid and sharp front at its edge without elongation of cell density profile into peripheral zone typical for soft matrixes, as on Figures 4 and 5. Instead, the poor accumulation of the invading cells out of the core spheroid shrinks to a narrow densified zone due to cell–cell adhesion and the matrix tension, forming another proliferative rim (Figure 8A). By day two shown on the figure, the major part of the first proliferative rim is located in the area of oxygen shortage and undergoes cell phenotype transition, while the small frontal proliferative rim is formed in the area of high oxygen content. By day three over 90% of the main proliferative rim turns hypoxic, as the oxygen level inside the core part drops below the transition threshold (Figure 8C).

A radial component of the total stress in cell–ECM system exhibits strong gradient on the inner side of the second proliferative rim (Figure 8B). The peak magnitude of the radial stress component in front of the core spheroid *σ*_Rtot_ ∼ *σ*_0_ is nearly 10 times higher than that formed at basic ECM stiffness (compare with Figure 6). The resulting radial force pushes all cells within this spherical layer in outer direction, so that it covers a distance of ∼0.1 mm within a day (Figure 8A and c). Cell–cell adhesion and rigid matrix keep this layer compact and rather thin. After three days of MTS growth the mechanics of displacement of this distant proliferative rim is being gradually changed. The inner force, pushing it out is decreasing. At the same time, the oxygen level behind the rim goes down below the threshold of cell phenotype transition (Figure 8D). As a result, only outer cells of this layer retain proliferative type. The other cells are turning hypoxic. This process creates a growing trace of hypoxic cells behind the rim (compare Figure 8C and D). The cell density in the proliferative layer is decreasing and its expansion is conditioned to great extent by the level of oxygen at its outer side, sustaining actively proliferating regime. The speed of the proliferative layer expansion is determined now by oxygen consumption rate, as the layer follows the shrinking outer oxygen zone with its concentration above the threshold level (Figure 8D). Certain amount of hypoxic cells goes ahead of the proliferative layer due to their high motility, but they still do not contribute into the layer displacement, as the reverse phenotype transition hypoxic- to -proliferative requires rater long residence time (≥96 h) in the area of high oxygen concentration (see Table 4).

It’s important to note that the simulated scenario does not have an immediate practical meaning. The distant subtumor layer has been observed in simulation within rather narrow range of ECM stiffness values at specific oxygen concentration profiles and boundary conditions at the domain borders. However, all these parameters, including oxygen diffusion coefficients, cell motility, are feasible and typical for glioma MTSs (see Tables 1, 2, 3 and 4). The observed subtumor layer is formed at the main tumor interface and grown away from the core spheroid. This scenario is generally consistent with earlier obtained results of *in silico* models and biological experiments [55], [101] demonstrates that the subpopulations of tumor cells are being formed by multiple cells of the main tumor surface and not as a result of hyper-proliferation of a distant single cell. However, the models exploited in Refs. [55], [101] do not take into account viscoelastic interaction between the two compressible fractions cells and ECM. The influence of microenvironment on cell dynamics is considered without mutual rheological affects. The formation of cell subpopulations has been simulated in earlier models as a result of circumferential morphological instability coming from the interplay between oxygen transport, cell phenotype transitions and cell adhesion. The current model demonstrates that the same processes together with effects of compressibility and mutual elastic influence in cell–ECM system may lead directly to radial discretization of invasive zone and formation of separate cell populations.

**Figure 9:**
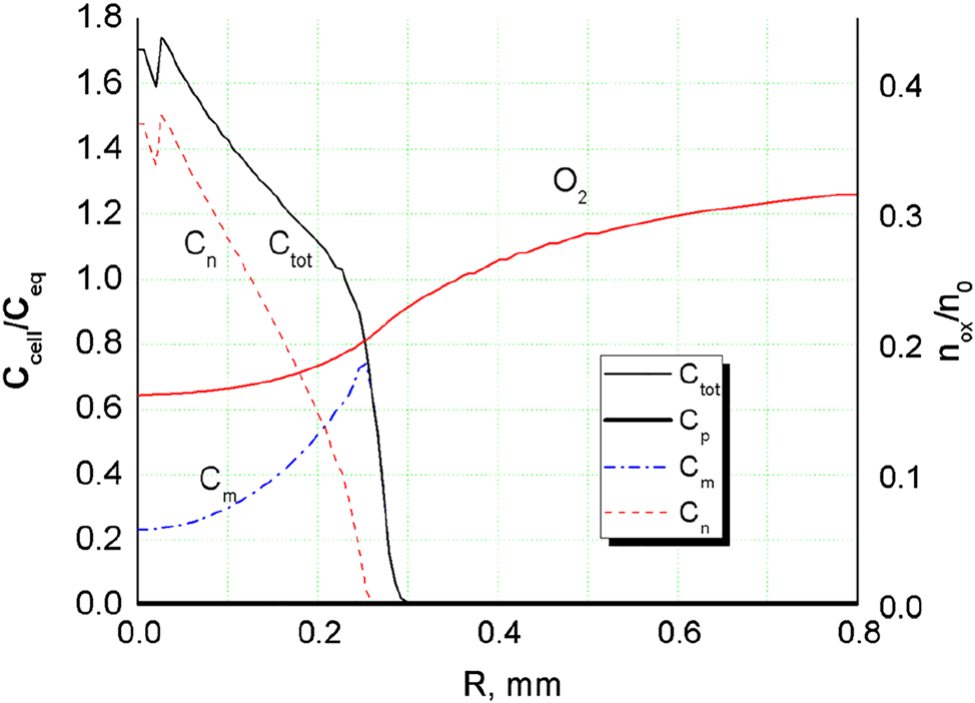
Calculations carried out for the same conditions as on Figure 8, except for limited oxygen volume in the domain. ECM stiffness *E*_ECM_ = 2.5 kPa, RBC for oxygen supply. Radial cell density distributions are presented for day 5: proliferative cells *C*_*p*_, hypoxic *C*_*m*_, necrotic *C*_*n*_, total cell density *C*_tot_; and distribution of oxygen concentration.

It is important to stress that permanent oxygen supply through the domain border (PSBC) plays a pivotal role in formation of subtumors. The basic boundary conditions for oxygen supply RBC, when the total amount of oxygen in the domain is limited to constant value, do not lead to formation of distant cell agglomerations out of the core spheroid (Figure 9). The calculation results for RBC and the same as above ECM stiffness *E*_ECM_ = 2.5 kPa show a sharp edge of the tumor spheroid and no invasive zone or distant cell clusters. By day 5 the proliferation rim disappears completely, as shown on Figure 9, due to general shortage of oxygen in the domain and its low level inside the spheroid in particular.

## 4 Discussion

A two component *in silico* model of glioma MTS growth, presented in this work takes into account rheological properties of cell–ECM system, as well as the effect of mechanical ECM deformation. Cell colony, as a separate component in the model represents a porous compressible aggregation. The model considers interaction between the two compressible constituents of the composite system, visco-elastic cell aggregation and elastic matrix, within the whole simulation domain.

The transitions between the three cell phenotypes, proliferative, hypoxic and necrotic, were triggered by the local oxygen content, which was the only metabolite considered in the model. It was demonstrated that diffusion of oxygen from the peripheral zone towards the spheroid center and its distributed consumption built a specific profile of oxygen content, which affects in a self-consistent loop the cell density profile and ultimately the character of tumor invasion.

Special consideration has been given to the case of reduced diffusion of oxygen, which sharpens spheroid edge, bringing the result more in line with the observed shapes, but generally reduces cell concentrations in the core part below the experimental values.

The simulated cell density profiles are in good quantitative agreement with these obtained in experiment within the major part of the spheroid, except for the spheroid center, where the simulated density is only 60% of the measured value. Cell densities measured in the center cannot be explained by local cell proliferation only, being well above the figures estimated in that simple way. Another factor affecting this magnitude is a densification of the center through its compression.

The initial contraction observed experimentally, as slight decrease of spheroid radius has been also obtained in simulation. As long as the starting cell concentration in the spheroid is below equilibrium value, so that cell–cell adhesion overcomes repulsive intercellular forces, the model demonstrates certain shrinkage of the initial tumor volume.

Further compression of cell aggregation in the central part of MTS due to the pressure exerted by expanded ECM has also been simulated. Even though, the simulated compression does not provide sufficient densification of the spheroid center to correspond exactly to the measured values.

An artificial increase of ECM stiffness above the values typical for collagens used in MTS growth tests elevates the calculated cell densities in the center much closer to the measured value, but still not high enough to match it exactly. The model presumably underestimates compressive forces, acting within the spheroid or compressibility of the given cellular continuum. These results demonstrate however, an important role of stresses and compressibility of cell-matrix composite system in MTS growth.

A relatively thin transition layer forms on the spheroid surface, where mechanical characteristics of cell–ECM composite undergo rapid transition. This layer is an important part of MTS structure and results from elastic ECM reaction on spheroid expansion. This structure can be simulated only within the two component model that takes into account the mutual influence of the two constituents of the composite system. Their interaction forms a specific cell density profile inside the spheroid and the Transition Layer on its surface. In particular, there is a nearly constant level of the total stress in the MTS core behind the transition layer. The flat level of tensile stress results from compensatory mechanical interaction between cell aggregation, which forms compressive stress and the stretched ECM with growing tensile stress. Outside the core spheroid, within transition layer ECM exhibits rapid relaxation of tensile stress and cell concentration quickly drops down resulting in sharp change of intercellular stress from compressive to tensile character due to cell–cell adhesion.

The simulations demonstrated that sharp stress relaxation within the transition layer should lead to a reordering of ECM structure. In the case of MTS growth in collagen, the fibers are expected to reorder in front of the Layer aligning to the spheroid surface, undergoing tensile tangential and compressive radial forces. Another reorientation of fibers is expected inside the spheroid, along the radius. This type of fiber reordering has been observed in MTS experiments [29] as mechanical remodeling of ECM. The matrix remodeling should have a modulatory effect on collagen permeability and therefore cell invasiveness. However, there is no clear quantitative model to take it into account.

Another effect of mechanical ECM remodeling through matrix deformation has been studied quantitatively in the current simulations. It was reasonably assumed that the cell population growing into ECM scaffold causes its expansion, stretching pores and therefore affecting its permeability and further-mobility of invasive cells. The average effect of this matrix deformation resulted in increase of tumor invasion speed up to 20%. This result demonstrates the importance of mechanical effects on ECM structure and their possible influence on cell invasiveness.

Rather brief consideration has been given in the work to the influence of particular physical factors, such as cell–cell adhesion, rheological properties of the matrix and cell aggregation, oxygen supply and diffusion rate on glioma mechanobiology. Each of these factors deserves closer study including *in silico* simulations of microscale scenarios.

The simulation results show sensitivity to the type of GoG model. The stiff GoG model, which does not allow any proliferation of hypoxic cells and vice versa, demonstrates relatively low cell density profile and steep edge of the MTS core. The overlapped GoG model shows overall higher cell density, more elongated profile and better agreement with the experimental results.

The simulated glioma spheroids exhibit higher cell density and faster expansion in rigid matrixes that is in line with *in vitro* studies of glioma MTSs [6], [19]. The model shows that an increase of ECM stiffness sharpens the edge of MTS, which tends to form compact structures in rigid matrixes. Wherein, the cell density profile is getting shorter and steeper due to decrease of invasive zone. The shrinkage of the invasive zone in rigid ECM is conditioned by high potential barrier for motile cells to travel across transition layer, against the background of large stress gradients across the surface of the core spheroid.

Two scenarios have been observed in simulations for rigid matrixes: either a single compact spheroid with sharp edge is being formed or the single spheroid is accompanied by additional fragmented cell structures instead of elongated continuous invasive zone of low cell density typical for soft matrixes. The simulations indicate a presence of specific range of ECM stiffness and nutrient concentrations when additional proliferative rim may grow away from the main one. A specific stress distribution forms hydrostatic radial pushing forces at the edge of MTS core spheroid. So, that the second proliferative rim is being pushed further away. This result demonstrates that cell motility is probably not the key factor in formation of distant subtumor clusters, as it would play an important role only in the scenario when a single highly motile cell could invade far into nutrient-rich area and hyper-proliferate there. This scenario did not take place in the current study. The further motion of the distant proliferative layer is provided by active proliferation in the outer zone reach of nutrients (oxygen in the current model). Cell–cell adhesion and ECM stiffness play crucial role in formation of these distant multicellular proliferative clusters.

Over all, three scenarios of glioma invasion at different ECM stiffness values, driven by mere mechanical principles of continuum and iso-strain dynamics of cell–ECM composite, have been shown in simulations. MTS growing in soft matrix exhibits gentle cell density profile with elongated low density invasive zone, while rigid ECM conditions sharp edge of MTS and higher invasion speed. Moreover, the highest speed of tumor invasion is obtained at intermediate values of ECM stiffness, where a compact cell clusters are being formed instead of invasive zone as additional proliferative layers out of the core spheroid.

It is important to mention that the current 1D model simulates spherically symmetric process of tumor growth. In a real 3D MTS growth, elongated protrusions in the form of fingers should form due to morphological instability of tumor border [55], [102], generating azimuthal discretization of cellular continuum.

In subtumor conditions, radial discretization of cell density profile is added to the azimuthal one. This case should exhibit random array of proliferating islands separated from the main tumor spheroid, instead of distant spherical layers obtained in 1D simulations, as the uniform layers must fall off due to azimuthal instability. This characteristic dispersed distribution of distant proliferative islands has been observed in experiments [32], [68]. These patterns were identified as ‘starburst’ type distributions, typical for aggressive glioma cell lines.

The scenarios considered in the current study are based completely on relatively simple mechanics of elastic compressible composite system. It demonstrates scenarios of tumor invasion that can be used as basic approximation in further analysis of avascular stage of glioma including its signaling context.

Quantitative calculations of the effects of mechanical ECM restructure was currently limited to alteration of pore sizes only, under stress imposed. Detailed simulations of matrix structure and its remodeling effects, which play substantial role in tumor progression, require further evolution of the model.

Another important factor of MTS growth not included into the model, that may have an appreciable effect on mechanobiological aspects of glioma growth is the removal of necrotic cells from the core spheroid. It may particularly affect cells and stress distributions inside the spheroid within relatively long growth times and is worth including into the model in its further development.
