# Supplementary material for: Cell – extracellular matrix interaction in glioma growth. I n silico model
Source: J Integr Bioinform. 2020 Dec 14:20200027. doi: 10.1515/jib-2020-0027 (PMC7849632; doi:10.1515/jib-2020-0027)
Supplement: Supplementary file 1 [file jib-17-20200027-s001.doc]

**Supplementary file to**

**Cell – extracellular matrix interaction in glioma growth**

***In silico* model**

Vladimir Kalinin

**Variations of Model and Parameters**

To illustrate the effect of ECM stresses on MTS growth a number of calculations were carried out with different matrix properties. The comparison of the results is shown on **figs. S1- S3**. The first case represents the single- constituent model, where no force is exerted from ECM on cell aggregation, so that the total stress in the composite system is *σ_tot_ = σ_C_* instead of (14). This case does not form any Transition Layer and the cell distribution differs dramatically from that obtained in vitro by Stein and colleagues (2007) and shows how important role the elastic ECM scaffold plays in MTS growth (**fig. S1**, no ECM case). The zone of high cell density in the spheroid center observed in experiment is being formed to great extent due to elastic stress in the matrix. So, once the elastic resistance of ECM is removed from the model, the cell density of the central part drops down dramatically.

Another case presented on **figure S1** shows day 3 cell distribution for permanent oxygen supply through the domain border (PSBC case). The total cell distribution is very close to what is shown on fig. 4 **of the main text**. Therefore, up to day 3 of MTS growth the general limit of oxygen in the closed domain, assumed in basic boundary conditions (RBC) and used for most calculations here does not have noticeable effect on MTS growth. In that initial period, the limiting factor of oxygen supply is only oxygen diffusion towards MTS. The above variations of oxygen supply in the model produce cell density distributions in the peripheral part of the spheroid very close to the one observed in experiment. At the same time, the cell density in the center has been always lower in simulations than the value observed *in vitro*. Once this value is substantially affected by ECM stiffness, another calculation has been carried out for the case of stiff matrix at Young’s Modulus E_ECM_= 5kPa in order to bring the calculated cell density in better correspondence with the experimental data. The resulting cell density distribution by day 3 is presented on **fig. S1**. The resulting cell densities in the core spheroid are higher than those obtained for the basic conditions, see the figure. The core spheroid exhibits faster expansion. ECM stresses and the compensatory hydrostatic intercellular pressure inside the spheroid are obviously higher than calculated in basic conditions, as the high ECM stiffness requires higher hydrostatic pressure to [maintain](https://www.thesaurus.com/browse/maintain) MTS expansion. The rigid ECM scaffold develops high stress gradients within TL, forming significant potential barrier for motile cells traveling out of the core spheroid. This condition keeps the spheroid compact reducing significantly the size of typical low density IZ.

The next series of calculations compared two different modes of cell phenotype switch for proliferation-to-hypoxic transition (**fig. S2**). The basic switch mode, when each of the two cell phenotypes may retain properties of the other type in limited extent (**see Table 1 in the main text**) is compared with stiff GoG rule, when proliferative cells do not show any motility, as well as no proliferation is allowed for hypoxic cell type. The stiff GoG model produces noticeably lower cell density over the whole inner part of the spheroid and a sharp profile of density distribution at the same time. In general, the stiff GoG conditions produce results very similar to the case with reduced oxygen diffusion presented on fig.5 **of the main text**.

**Use Figure S1**

**Use Figure S2**

The **fig. S3** illustrates MTS growth in a situation when ECM permeability is a function of its stress according to (4). This is the situation when ECM structure is partially affected by stress distribution within one particular aspect- its porosity. The stretching effect of tensile forces leads to increase of average pore size and consequently - matrix permeability. The calculation shows that this effect stimulates tumor cell invasion resulting in faster MTS growth. The average increase of the growth speed is about 20% (**fig. S3**).

**Use Figure S3**

(**Stein et al, 2007**) Stein A, Demuth T, Mobley D, Berens M, Sander L. A mathematical model of glioblastoma tumor spheroid invasion in a three-dimensional in vitro experiment. Biophysical Journal. 2007 Jan; 92(1):356–365; DOI: [org/10.1529/biophysj.106.093468](https://doi.org/10.1529/biophysj.106.093468)

| 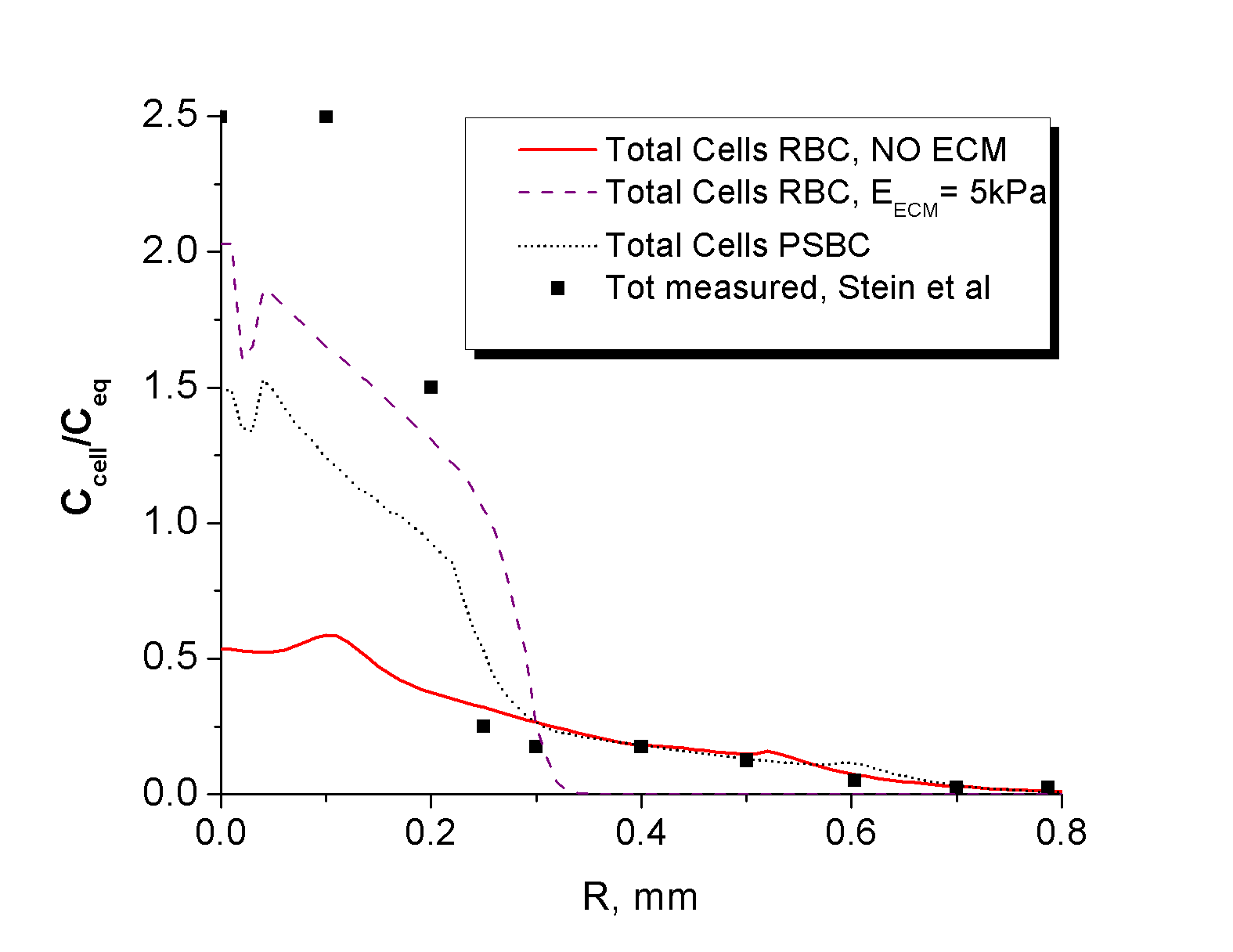 |
| --- |
| **Figure S1:** Day 3 of MTS growth at different conditions. Solid red line illustrates distribution calculated within a single constituent, where no effect from stressed ECM on cell dynamics is taken into account, *σ_tot_ = σ_C_*. As a result, no TL has been formed and cell density profile does not exhibit dense area in the middle. The same basic conditions of MTS growth were applied in calculations, as used for the results presented on figs. 5-7. The dotted line shows cell density calculated at basic conditions except for oxygen supply, which was provided through PSBC. Both cases exhibit very similar cell density profiles in the spheroid periphery.  Very stiff ECM E_ECM_= 5kPa conditions, higher densification in the middle and lack of peripheral low density area (dashed line), which also does not correspond to the observed distribution by Stein et al. In contrast to the first two cases, this one does not show typically extended invasive zone. Instead, cell density profile exhibits a sharp drop of the value down to zero. |

| 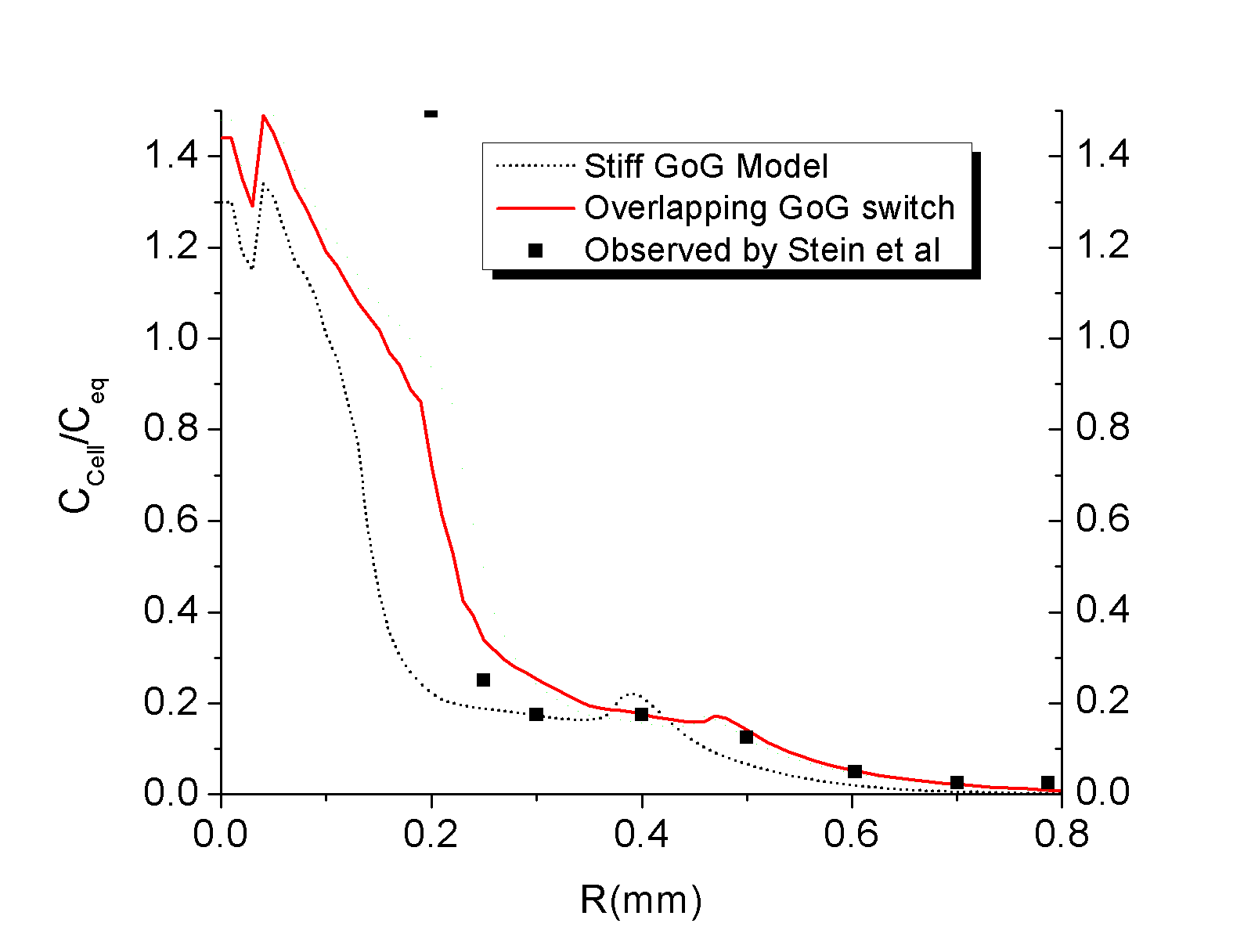 |
| --- |
| **Figure S2:** Cell density distributions for day 3 of MTS growth at basic conditions. Stiff GoG rule in comparison with the basic overlapping cell phenotype switch model. |

| 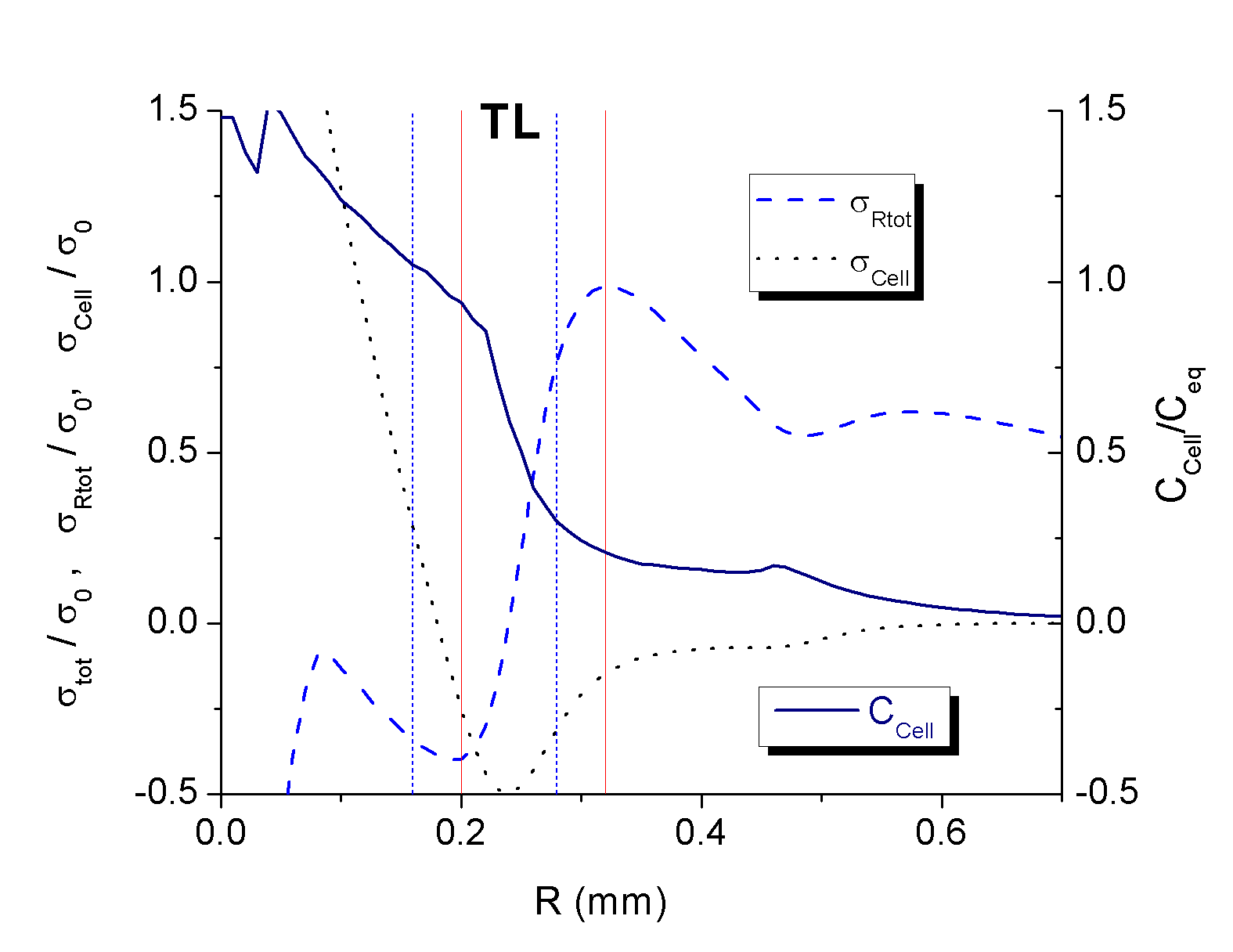 |
| --- |
| **Figure S3:** Stress distribution along MTS radius at day 3 with stress dependent ECM permeability. Vertical solid red (in colour version) show TL and dotted blue lines show TL position for the same moment of time without stress dependent ECM permeability as on fig.6; *σ_0_*= 100Ps. The shapes of stress and cell density distributions are generally very similar for both cases. However, stress dependent ECM permeability provides in average up to 20% faster MTS growth. |
